# Recent advances in cancer nanomedicine: From smart targeting to personalized therapeutics - pioneering a new era in precision oncology

**DOI:** 10.1016/j.mtbio.2025.102660

**Published:** 2025-12-10

**Authors:** Ayesha Younas, Shuanghu Wang, Muhammad Asad, Abdullah Al Mamun, Saadat Majeed, Ali Sharif, Quan Zhou, Yunxiao Liu, Peiwu Geng, Chuxiao Shao, Jian Xiao

**Affiliations:** a Key Laboratory of Joint Diagnosis and Treatment of Chronic Liver Disease and Liver Cancer of Lishui, Central Laboratory of The Lishui Hospital of Wenzhou Medical University, The First Afffliated Hospital of Lishui University, Lishui People’s Hospital, Lishui, Zhejiang 323000, China; b Oujiang Laboratory (Zhejiang Lab for Regenerative Medicine, Vision and Brain Health), School of Pharmaceutical Sciences, Wenzhou Medical University, Wenzhou, Zhejiang, 325000, China; c Department of Inorganic Chemistry, University of Chemistry and Technology Prague Technická 5, Prague, 616628, Czech Republic; d Institute of Chemical Sciences, Bahauddin Zakariya University, Multan, 60800, Pakistan; e Department of Pharmacology, Institute of Pharmacy, Faculty of Pharmaceutical and Allied Health Sciences, Lahore College for Women University, Lahore, Pakistan

**Keywords:** Stimuli-responsive nanoparticles, Omics enhanced nanomedicine, Artificial intelligence, Personalized nanomedicine, Tumor microenvironment (TME), Translational challenges

## Abstract

Cancer nanomedicine has evolved from the 1995 landmark approval of Doxil® into a programmable platform of precision oncology. The field now progresses along a coherent continuum that begins with passive enhanced permeability and retention (EPR)-mediated tumor accumulation, advances to active ligand–receptor targeting, and culminates in stimuli-responsive carriers whose cargo is liberated only when triggered by endogenous (acidic pH, redox imbalance, elevated GSH, dysregulated enzymes, ROS) or exogenous (light, magnetic, ultrasound, X-ray, electric) cues intrinsic to the tumor microenvironment (TME). This review maps this continuum, highlighting how the integration of patient-specific multi-omics data with artificial intelligence (AI) is converting tumor heterogeneity into quantitative design rules for nanocarrier optimization, validated in patient-derived organoids. Despite over 15 FDA-approved cancer nanomedicines and a robust clinical pipeline, translation is impeded by biological barriers, protein corona-mediated toxicity, manufacturing scalability issues, and a fragmented regulatory landscape. To bridge this bench-to-bedside chasm, we propose a convergent roadmap: safe-by-design engineering, quality-by-design modular manufacturing, and AI-guided digital twins coupled with micro/nano-robotic delivery for real-time, adaptive dosing. Realizing this vision will transform nanomedicine from an empirical carrier technology into a patient-calibrated, closed-loop therapeutic engine, cementing its role as the frontline of precision oncology.

## Graphical abstract

**Figure:**
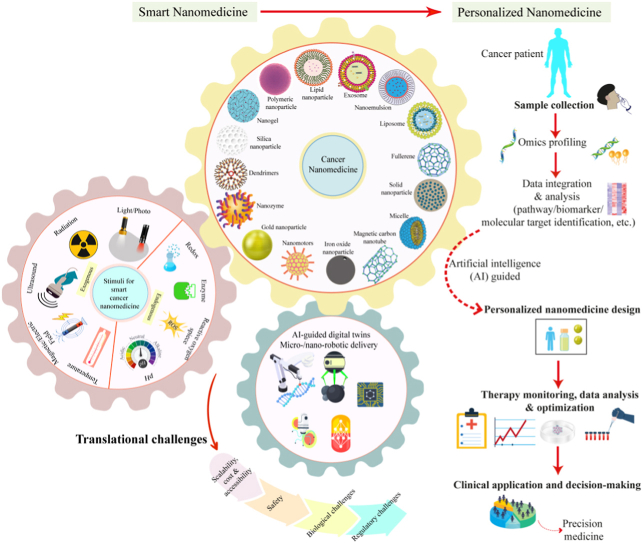

## 1 Introduction

Cancer continues to outpace most lethal diseases, claiming nearly 10 million lives in 2022, roughly one death every 3.2 s, while adding 20 million new diagnoses to an already strained global healthcare system [1]. If current demographic and lifestyle trajectories persist, annual incidence is projected to surpass 26 million cases and 17 million deaths by 2030, with the steepest rises expected in low- and middle-income regions. These figures are not merely statistics; they reflect the compounded impact of ageing populations, shifting dietary patterns, environmental carcinogens, and lifestyle transitions [2]. These trends underscore the urgent need for therapeutic innovations to curb mortality without imposing unsustainable toxic burdens. Current standard-of-care modalities, such as surgery, radiotherapy, chemotherapy, molecularly targeted therapy, and immunotherapy, have undoubtedly improved survival; yet, they remain encumbered by intrinsic limitations [[3], [4], [5]]. Localized interventions such as surgery and radiotherapy achieve curative intent only when the disease is anatomically confined, yet more than half of patients relapse or develop distant metastases [6,7]. Systemic therapies, the mainstay for disseminated disease, are hindered by two intertwined problems: off-target toxicity and inter-patient heterogeneity. Chemotherapeutic agents, for example, indiscriminately attack rapidly dividing cells, producing dose-limiting neutropenia, neuropathy, and cardiotoxicity. Immunotherapies generally achieve objective responses in approximately 15–30 % of unselected patients with solid tumors [8]. Photodynamic, photothermal, and gene-based therapies hold significant potential but are still in the process of overcoming clinical translational challenges, including limited tissue penetration, vector immunogenicity, and manufacturing complexity. Furthermore, in numerous cases, the late-stage presentation and rapid enzymatic or renal clearance of biologics, such as mRNA, monoclonal antibodies, and recombinant proteins, continue to erode the efficacy of conventional regimens [[9], [10], [11], [12]]. Consequently, the therapeutic window narrows, survival gains plateau, and off-target toxicities escalate. To break this impasse, oncology is shifting from empirical “one-size-fits-all” protocols to precision paradigms that tailor interventions to the molecular anatomy of each tumor and the pharmacological landscape of each patient [13,14]. Nanomedicine has emerged as the enabling platform for this transition, uniting nanotechnology, biomaterials science, and pharmaceutical engineering to stabilize payloads in circulation, navigate complex biological barriers, and execute tumor microenvironment (TME)-triggered release. Over the past three decades, this convergence has evolved from first-generation liposomes to sophisticated, multi-stimuli-responsive nanocarriers that co-deliver cytotoxins, immunomodulators, and gene-editing cargoes, converting systemic liabilities into programmable therapeutic opportunities [[15], [16], [17]]. Nanomedicine aims to sharpen dose-response curves, raise systemic bioavailability, and direct drugs to their intended cellular targets, thereby improving cancer outcomes [18]. A versatile collection spanning lipid-based liposomes, inorganic superparamagnetic iron oxide nanoparticles (SPION), and polymeric micelles has been engineered to transport nucleic acids, checkpoint inhibitors, and cytotoxic drugs deep into malignant tissue [19,20]. Regulatory milestones underscore this translational momentum; at least 15 nanomedicines have been approved worldwide, while more than 80 successors are navigating over 200 active clinical trials. Notably, all approvals rely on passive targeting via the enhanced permeability and retention (EPR) effect; to date, not a single actively ligand-directed construct has reached the market, although a handful are progressing through early-phase evaluation [18]. This failure to translate actively targeted designs to the clinic highlights a significant gap between preclinical promise and clinical efficacy. It exemplifies a broader trend of high attrition, where even nanocarriers engineered with sophisticated targeting ligands and stimuli-responsive mechanisms are frequently thwarted by the complex biological realities of human tumors and the challenges of scalable manufacturing. In direct upshot to these translational barriers, the field is increasingly turning towards a more nuanced, data-driven approach. Advances in tumor biology and nano-bio interface science have catalyzed the emergence of personalized nanomedicine whose size, charge, and surface chemistry are tuned to individual tumor profiles. These next-generation systems concentrate drug payloads within the tumor bed, thereby reducing systemic exposure and improving therapeutic indices [21,22]. Yet the leap from bench to bedside remains steeper than for conventional drugs. Biological barriers, scalability bottlenecks in manufacturing, accessibility concerns, and cost-effectiveness issues collectively erode the competitive edge of nanotherapeutics. Future efforts must prioritize post-administration stability, patient-specific design algorithms, reproducible scale-up protocols, and rigorous, safety-by-design physicochemical characterization to unlock their clinical potential [23,24].

Building on these premises, the present review is structured to provide a rational framework that traverses the entire translational arc of cancer nanomedicine from fundamental targeting concepts to clinical deployment and future innovation. We first dissect the hierarchical targeting strategies (passive EPR, active ligand–receptor engagement, multi-stimuli-responsive release, and bio-responsive) and benchmark their performance against evolving tumor biology. We then integrate multi-omics datasets and machine-learning (ML) pipelines to demonstrate how patient-specific molecular signatures can be translated into quantitative design rules for the size, charge, surface chemistry, and stimulus sensitivity of nanocarriers and can be validated by organoid-based models. Subsequent sections critically evaluate the current clinical landscape, highlighting approved nanomedicines and analyzing the extensive portfolio of over 200 active trials, alongside notable failures, to illuminate the key challenges that contribute to high attrition rates. Furthermore, we expound the key bottlenecks underpinning this translational gap, including biological barriers and safety concerns, translational hurdles in scalable manufacturing and cost-effectiveness, and a lack of harmonized regulatory frameworks. Finally, we propose a convergent roadmap that couples safe-by-design engineering, AI-guided formulation, decorous organoid-based validation, and micro-/nano-robotic delivery to transform nanomedicine into a programmable therapeutic platform. This delineates the scientific and translational imperatives required to evolve nanomedicine from an empirical carrier technology into a data-driven, patient-calibrated therapeutic modality capable of matching tumor heterogeneity with programmable precision, fulfilling its mandate as the frontline of precision oncology.

## 2 Cancer nanomedicine

**Fig. 1:**
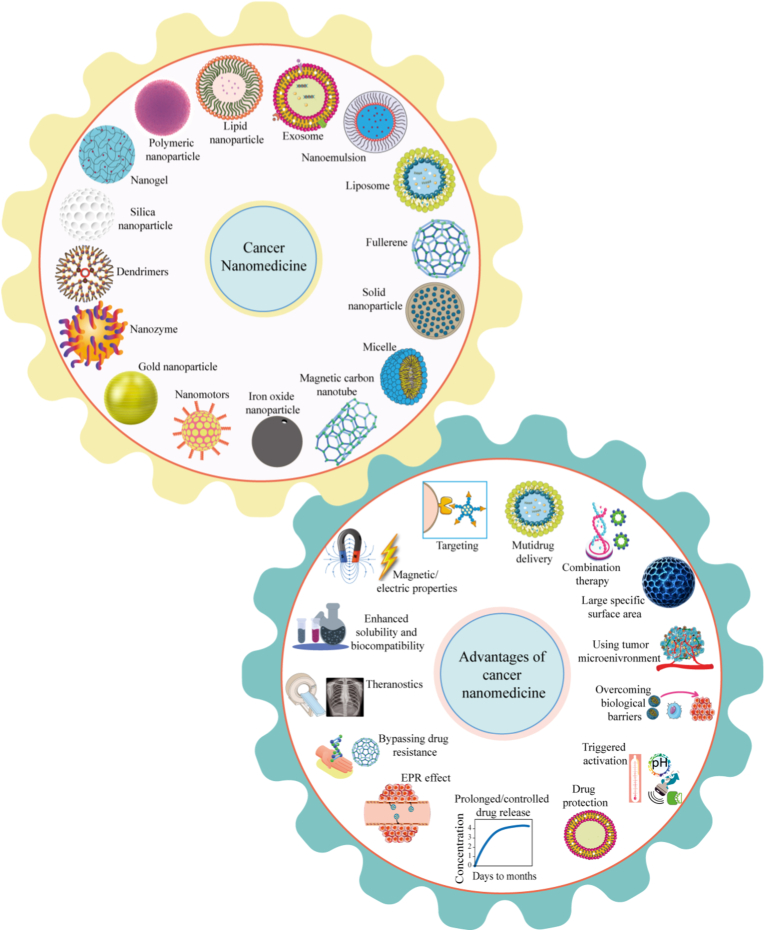
Overview of selective nanomedicine-based drug-delivery systems and their advantages over conventional approaches for cancer therapy. (EPR– Enhanced Permeability and Retention).

Cancer nanomedicine (Fig. 1) has emerged as a transformative convergence of nanotechnology and medical science, reshaping oncological treatment paradigms. By exploiting the unique physicochemical attributes of nanoscale constructs typically 1–100 nm, researchers have developed a versatile nanoparticle that includes liposomes, polymeric micelles, dendrimers, mesoporous silica, and metallic or magnetic nanoparticles. These carriers possess exceptional advantages (Fig. 1), including surface area-to-volume ratios, which enable the high payloads of chemotherapeutics, nucleic acids, or imaging agents while shielding healthy tissues. Consequently, nanomedicine can amplify intratumoral drug concentrations through passive (EPR-mediated) or active (ligand-directed) accumulation, thereby minimizing off-target toxicities such as neutropenia, alopecia, and immunosuppression [18,[24], [25], [26]]. Beyond simple delivery, stimuli-responsive nanomedicine can sense the acidic pH, redox gradients, enzymatic milieu, or hypoxia intrinsic to the TME, triggering on-demand cargo release with spatiotemporal precision [27,28]. This programmable nanopharmacology simultaneously expands the therapeutic index of conventional cytotoxics and consolidates multimodal interventions, such as chemotherapy, gene therapy, photothermal ablation, and immune checkpoint modulation, into a single, integrated nanoplatform. By leveraging patient-specific multi-omics signatures, theranostic nanomedicine is now rationally engineered to co-localize targeting ligands, therapeutic payloads, and imaging reporters within a unified architecture. Systematic modulation of nanoparticle hydrodynamic diameter, surface charge, and stealth coating density enables organotropic response, such as galactose-decorated nanocarriers that exploit asialoglycoprotein receptor (ASGPR)-mediated clathrin-dependent endocytosis for selective hepatocellular accumulation [29], whereas CD31/PECAM-1-targeted vectors undergo endothelial transmigration to traverse blood–brain or pulmonary microvasculature with molecular precision [30]. Delivery modalities are further diversified via intranasal mucosal deposition, dissolvable microneedle-mediated transdermal flux, intratumoral depot injection, or oral nanocrystal formulations, etc., each exploiting distinct anatomical gateways to maximize local drug exposure while minimizing off-target systemic toxicity.

## 3 Cancer nanomedicine-based smart targeting

### 3.1 Active and passive targeting

**Fig. 2:**
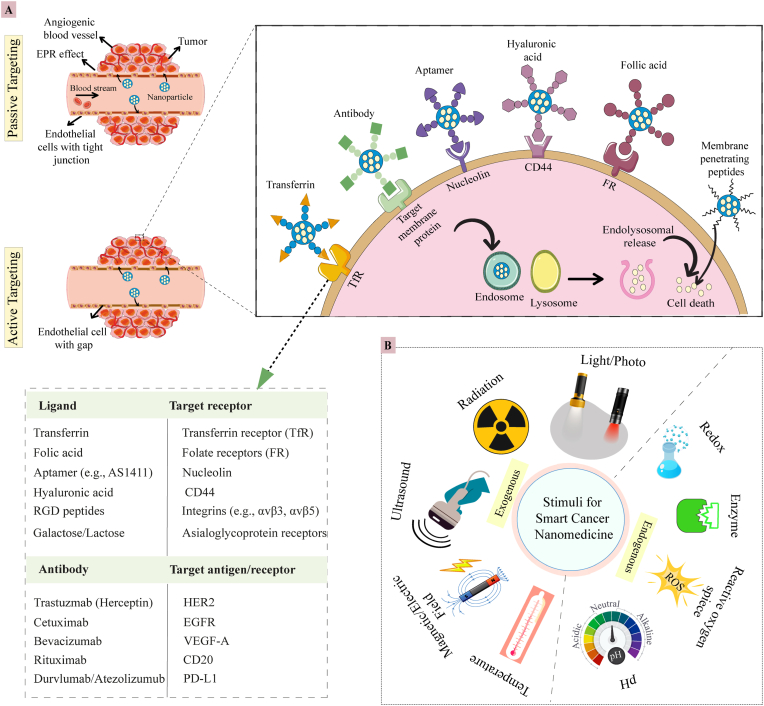
**(A)** Illustration of passive targeting of nanomedicine via the enhanced permeability and retention (EPR) effect, and active targeting via ligand- or antibody-conjugated nanoparticles. **(B)** Exogenous and endogenous stimuli in smart cancer nanomedicine delivery. (HER2 - Human epidermal growth factor receptor 2, EGFR - Epidermal growth factor receptor, VEGF-A - Vascular endothelial growth factor A, CD20 - Cluster of differentiation 20, PD-L1 - Programmed death-ligand 1, CD44^−^cell surface glycoprotein).

Nanomedicine employs passive and active targeting strategies to deliver drugs to tumors (Fig. 2A). Passive targeting exploits the tumor-selective EPR effect, whereby nanoparticles extravasate through leaky neovasculature and are retained by dysfunctional lymphatic drainage, concentrating the payload at the tumor site while sparing normal organs. The magnitude of EPR-mediated accumulation is primarily dictated by carrier diameter (10–200 nm), zeta potential (e.g. ±10–30 mV), and shape (e.g. sphere vs rod vs disc vs worm-like), rather than by molecular recognition. This size range avoids renal clearance and reduces uptake by tissue-resident macrophages, while allowing nanoparticles to pass through endothelial openings [18,31]. However, the EPR effect is highly heterogeneous, varying significantly across tumor types, stages, and individual patients, limiting the universality of this approach [32]. It is more pronounced in soft-tissue sarcomas than in dense pancreatic ductal adenocarcinoma. Therefore, merely designing a nanoparticle to exploit EPR is an insufficient strategy. The field must now pivot towards adapting to this heterogeneity, which can be achieved through two complementary avenues: Patient stratification using EPR-imaging companions (e.g., ferumoxytol MRI or radiolabeled liposomes) to identify likely responders, and the development of dynamic nanomedicines that can actively enhance their own delivery, for instance, by co-administering vessel-priming agents (e.g., radiotherapy) to improve vascular permeability in a patient-specific manner, transiently [[33], [34], [35]]. Alongside these adaptive strategies, active targeting provides a more direct approach to circumvent the limitations of passive EPR. By functionalising nanoparticles with ligands, antibodies, peptides, aptamers, or small molecules that engage receptors overexpressed on malignant cells or tumor-associated vasculature, active targeting aims to achieve specificity independent of vascular permeability. Upon binding, receptor-mediated endocytosis delivers the cargo directly into the cytosol, increasing intracellular drug levels and circumventing efflux pumps. Efficacy, however, depends on receptor abundance; antigen-negative subclones can escape treatment. To overcome this, researchers are developing multivalent constructs bearing two or more distinct ligands that recognize complementary receptors, thereby widening the target spectrum and improving binding avidity. Implementation of such strategies requires detailed molecular profiling of each tumor to ensure ligand-receptor compatibility and minimize off-target effects. Although this method enhances precision and reduces the likelihood of off-target effects, it requires a detailed understanding of the tumor's molecular profile [36,37].

### 3.2 Stimuli-responsive targeting

Beyond ligand-directed accumulation, nanomedicine increasingly exploits the TME itself as a biological trigger for precision release [28]. There are mainly two kinds of stimuli for smart delivery of cancer nanomedicine (Fig. 2B). The endogenous stimulus-responsive nanoparticles trigger as the mildly acidic extracellular pH (6.5–6.9), millimolar GSH concentrations, tumor-enriched proteases, or elevated hydrogen peroxide (H_2_O_2_) induce structural collapse or chemical cleavage of specially designed linkers (hydrazone, disulfide, peptide), ensuring that the payload is freed exclusively within malignant cells [[38], [39], [40], [41]]. The exogenous stimuli-responsive nanoparticles are triggered by near-infrared (NIR) light, ultrasound, alternating magnetic fields, ionizing radiation, or electrical pulses, providing orthogonal, operator-controlled activation. Photothermal liposomes, magnetically heated iron oxide cores, ultrasound (US)-rupturable vesicles, and X-ray-activated scintillators exemplify platforms that release drugs or generate cytotoxic heat/ROS, on demand, independent of tumor heterogeneity. By integrating endogenous and exogenous stimuli, next-generation nanomedicines are evolving into “logic-gated” delivery systems that require dual or sequential signals for activation, thereby maximizing tumor specificity while preserving the integrity of healthy tissue [28,42,43].

#### 3.2.1 Endogenous stimuli-responsive nanoparticles

##### 3.2.1.1 pH-responsive nanoparticles

**Fig. 3:**
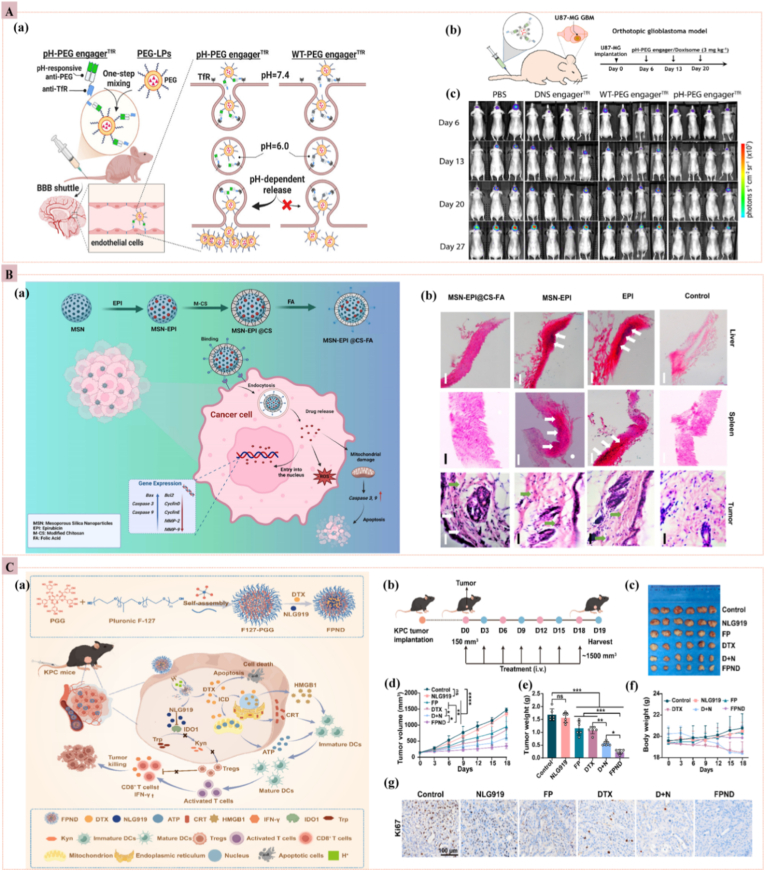
**(A)** Multimodal, pH-triggered nanomedicine (a) Schematic presentation of a pH-cleavable PEG “engager” that binds both TfR on brain endothelium and PEGylated nanodrugs, enabling blood-brain-barrier shuttling at pH 6.0 and release at pH 7.4. (b) Anti-GBM (Glioblastoma) action therapy timeline: Doxisomes coated with the pH-PEG engager^TfR^ complex. (c) *In vivo* efficacy: Orthotopic U-87 MG-Luc2 GBM mice received weekly i.v. injections. Injections (3 mg kg^−1^) of PBS, DNS-engagerTfR, WT-PEG-engager^TfR^, or pH-PEG-engager^TfR^-decorated Doxisome, and tumor growth was tracked by weekly IVIS bioluminescence imaging [49]. **(B)** (a) MSN-EPI@CS-FA: mesoporous silica nanoparticles loaded with epirubicin (EPI), surface-coated with chitosan–folate for tumor-specific uptake. (b) H&E staining of mice treated with EPI, MSN-EPI, and MSN-EPI@CS-FA on day 32; Scale bar: 200 μm [50]. **(C)** (a) FPND nanoparticle synthesis and function: IDO1 inhibition plus ICD to reprogram the immunosuppressive PDAC microenvironment. (b) Timeline of orthotopic KPC tumor implantation and treatment regimen. (c) Excised tumors after indicated therapies. (d) Tumor growth curves. (e) Final tumor weights. (f) Mouse body-weight changes. (g) Ki67 staining after treatment (Scale bar: 100 μm) [51].

pH-responsive nanoparticles, engineered to react to the acidic conditions of both the extracellular TME and intracellular compartments, represent one of the most extensively studied strategies in targeted cancer therapy. This approach leverages the steep proton gradient established by solid tumors, enabling precise and site-specific drug release. Even under aerobic conditions, malignant cells up-regulate glycolytic flux and convert up to 85 % of incoming glucose to lactate (the Warburg effect), dropping extracellular pH to 6.5–7.0, whereas endosomes (≈5.0–6.5) and lysosomes (≈4.5–5.0) remain markedly acidic [44,45]. This pH gradient forms the foundation for pH-responsive nanomedicines, which operate through two complementary mechanisms. The first involves acid-labile linkers (e.g., hydrazones, vinyl esters, orthoesters, and amides) that selectively hydrolyze under tumor-relevant acidic conditions, releasing covalently bound drugs within the ECM or acidic vesicles. The second utilizes charge-reversal polymers containing tertiary amines or carboxylates that protonate at low pH, shifting surface charge from neutral or negative to positive. This enhances electrostatic attraction to the negatively charged tumor cell membranes, facilitating endosomal escape [46,47]. To further exploit this differential, pH-tunable nanocarriers are often designed to undergo rapid surface charge switching in response to acidic pH. While in circulation (pH ∼7.4), they typically display a neutral or slightly anionic surface, which minimizes serum protein adsorption and prolongs systemic circulation. Upon reaching the acidic tumor interstitium (pH 6.5–6.9), acid-cleavable masking groups detach, revealing cationic moieties that invert surface charge. This promotes electrostatic interactions with cancer cell membranes, thereby accelerating cellular uptake. Once internalized, further protonation within endosomes destabilizes the vesicular membranes, promoting the cytosolic release of the therapeutic cargo and thereby enhancing drug efficacy while minimizing off-target toxicity to healthy tissues [48]. An innovative approach to pH-responsive drug delivery involves the use of bispecific antibodies to facilitate the targeted transport of polyethylene glycol (PEG)-based nanocarriers across the blood–brain barrier (BBB). In a recent study, researchers developed a pH-sensitive anti-PEG × anti-transferrin receptor (TfR) bispecific antibody, which can associate with PEG-modified nanoparticles at physiological pH and release them in acidic endosomal compartments during receptor-mediated transcytosis (Fig. 3A). This pH-triggered dissociation enables the effective detachment of the nanocarrier once it is inside the brain endothelium, thereby enhancing drug penetration into brain tissue. When tested in a glioblastoma (GBM) mouse model, the strategy enhanced the delivery of PEGylated liposomal DOX and prolonged survival, highlighting its potential for treating central nervous system malignancies. The key innovation lies in the structure-guided engineering of pH-responsive binding domains, which allow the antibody to switch its affinity depending on the surrounding pH. It offers a controlled and precise release mechanism that could be applied to various central nervous system (CNS)-targeted therapies [49]. *Kaveh-Farsani* et al. designed epirubicin (EPI)-loaded mesoporous silica nanoparticles (MSN) enveloped by chitosan (CS) and folic acid (FA), termed as MSN-EPI@CS-FA (Fig. 3B). The formulation leverages the acidic tumor environment to induce CS swelling, thereby doubling the rate of drug release at pH 5.4 compared to physiological pH, while folate-receptor targeting concentrates the payload in breast cancer cells. Achieving 79 % drug loading and negligible off-target accumulation, MSN-EPI@CS-FA markedly elevated pro-apoptotic markers and oxidative stress in MCF-7 cells and 4 T1 cell-induced tumors, underscoring its promise as a tumor-selective, pH-responsive nanotherapy [50]. *Li* et al. engineered a polyphenol-based pH-responsive nanoparticle (FPND) that co-encapsulates docetaxel (DTX) and the IDO1 inhibitor NLG919 through a one-step self-assembly process using Pluronic F127 and pentagalloylglucose (PGG). In the acidic microenvironment of pancreatic ductal adenocarcinoma (PDAC), FPND disassembles, releasing DTX to trigger potent immunogenic cell death (ICD) and NLG919 to block IDO1-mediated kynurenine production. This dual chemo-immunotherapy remodels the suppressive tumor microenvironment, enhancing CD8^+^ T-cell infiltration, reducing Treg recruitment, and suppressing tumor growth in KPC mice without inducing overt systemic toxicity (Fig. 3C) [51]. Despite the compelling rationale for targeting the acidic TME, the clinical translation of pH-responsive nanomedicines faces significant hurdles. A primary issue is the shallow and heterogeneous nature of the extracellular pH gradient. The often <1 pH unit differential from physiological conditions results in slow, incomplete drug release kinetics, while significant inter- and intra-tumoral variability means this trigger is not universally present. Compounding this, the most acidic regions are frequently hypoxic and poorly perfused, creating a biological paradox where the strongest stimulus exists in the areas least accessible to nanocarriers. This has prompted a reconsideration of the primary targeting mechanism, with a growing consensus that pH-sensitive chemistry may be more effectively utilized for ensuring endosomal escape, where the pH is more pronounced and reliable. The additional limitations include the circulatory instability of acid-labile linkers, risking off-target release, and the manufacturing complexity of these sophisticated systems, hindering scalable production [46,52].

##### 3.2.1.2 GSH-activatable redox-responsive nanoparticles

**Fig. 4:**
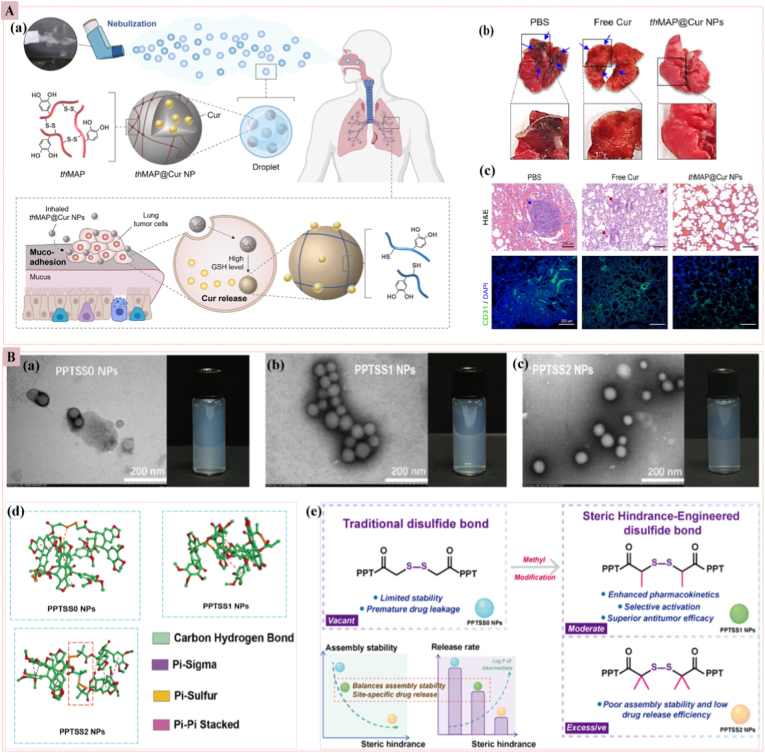
**(A)** Redox-responsive nanomedicine (a) Nebulized thMAP@Cur NPs release curcumin (Cur) via GSH-triggered disulfide cleavage in cancer cells. (b) Optical images of lungs with metastatic nodules (blue arrows). (c) H&E and CD31 staining showing reduced tumor burden [59]. **(B)** (a–c) TEM of different PPT nanoprodrugs; (d) Docking snapshots highlighting monomethyl steric tuning for optimal activation; (e) Steric-hindrance scheme comparing unsubstituted (−SS0-), monomethyl (−SS1-), and dimethyl (−SS2-) disulfide linkers, indicating balanced release vs. premature or sluggish activation [60]. (For interpretation of the references to color in this figure legend, the reader is referred to the Web version of this article.)

Redox-responsive nanoparticles exploit the striking GSH imbalance that distinguishes the TME from healthy tissue: intracellular GSH concentrations in cancer cells reach 2–10 mM, approximately four-fold higher than normal cells and roughly three orders of magnitude above extracellular levels. This gradient is leveraged to engineer nanocarriers containing disulfide, diselenide, succinimide-thioether, or trimethyl-locked benzoquinone linkers that undergo rapid reductive cleavage upon exposure to GSH, triggering site-specific drug release. Among these, disulfide-centered architectures remain the most extensively studied. Liposomes, polymeric micelles, mesoporous silica nanoparticles, and protein conjugates have all been functionalized with S–S bridges positioned either in the backbone or as core/shell cross-linkers [41,42,[53], [54], [55]]. For example, camptothecin and paclitaxel tethered to hydrophilic segments via S–S or Se–Se bonds exhibit accelerated liberation under high-GSH conditions, markedly enhancing intracellular cytotoxicity while minimizing systemic toxicity. Diselenide bonds, though less stable, offer faster cleavage kinetics [56]. Beyond simple release, manganese-dioxide-coated mesoporous silica systems synergize GSH depletion with Fenton-like metal-ion generation: intratumoral GSH reduction simultaneously ruptures disulfide gates for on-demand drug liberation and amplifies oxidative stress, thereby alleviating hypoxia and potentiating downstream antitumor immunity [57,58]. Inspired by the mussel's antioxidant chemistry, *Jeong* et al. recently engineered thiolated mussel-adhesive protein nanoparticles (thMAP NPs) that combine redox-triggered disulfide cleavage with mucoadhesive pulmonary retention. Thiolated MAP (thMAP) was cross-linked with oxidized glutathione (GSSG) to form disulfide-stabilized NPs (∼188 nm) that remain intact in the lung's oxidative milieu but specifically disassemble in cancer cells, where intracellular GSH was ≈ 2–10 mM (∼1000 × higher than plasma). Curcumin (Cur)-loaded thMAP NPs (thMAP@Cur NPs) exhibited 78 % drug release in 40 days under 10 mM dithiothreitol (DTT) (GSH mimic), yet <5 % leakage at physiological (10 μM) DTT, confirming cancer-exclusive activation. Inhaled thMAP@Cur NPs halve metastatic lung tumor burden in B16F10 mice vs. free Cur, with 10-day lung-selective retention and no systemic toxicity, demonstrating how mussel-inspired redox chemistry enables non-invasive, GSH-gated lung cancer therapy (Fig. 4A) [59]. *Liu* et al. constructed podophyllotoxin (PPT) homodimeric prodrug nanoassemblies in which the disulfide bridge is flanked by zero, one, or two methyl groups (−SS0-, −SS1-, −SS2-) to tune steric hindrance around the redox-labile bond. PEGylated nanoparticles (∼100 nm, >60 % drug loading) were stable in plasma, yet only the monomethylated −SS1- variant achieved the desired balance: robust circulation (AUC 3.25-fold higher than free PPT) and rapid cleavage in 1–3 mM DTT or 15–30 mM H_2_O_2_, releasing 37 % PPT in 24 h versus 1.5 % for −SS2- and 52 % for −SS0-. In 4T1 tumor-bearing mice, −SS1 NPs accumulated 4-fold in tumors, reduced tumor volume by 70 %, and eliminated the systemic toxicity observed with free PPT. This study demonstrates that judicious steric engineering of disulfide linkers can uncouple systemic stability from GSH-triggered activation, offering a refined redox-responsive nanoprodrug platform (Fig. 4B) [60]. Despite elegant GSH-gated activation, these nanosystems confront a logical triad of limitations: (i) circulating GSH (∼2–20 μM) can pre-reduce surface-exposed disulfides, leading to premature payload leakage before tumor contact; (ii) patient-to-patient and inter-tumoral GSH concentrations vary dramatically (spanning low millimolar ranges), so a single cleavage threshold cannot guarantee uniform drug release; and (iii) co-administered thiol supplements (e.g., N-acetylcysteine) can acutely raise systemic GSH, unpredictably accelerating off-target drug liberation and narrowing the therapeutic index [61,62]. Until real-time intratumoral GSH sensing is integrated, redox-responsive carriers remain hostage to this biological variability.

##### 3.2.1.3 Enzyme-responsive nanoparticles

**Fig. 5:**
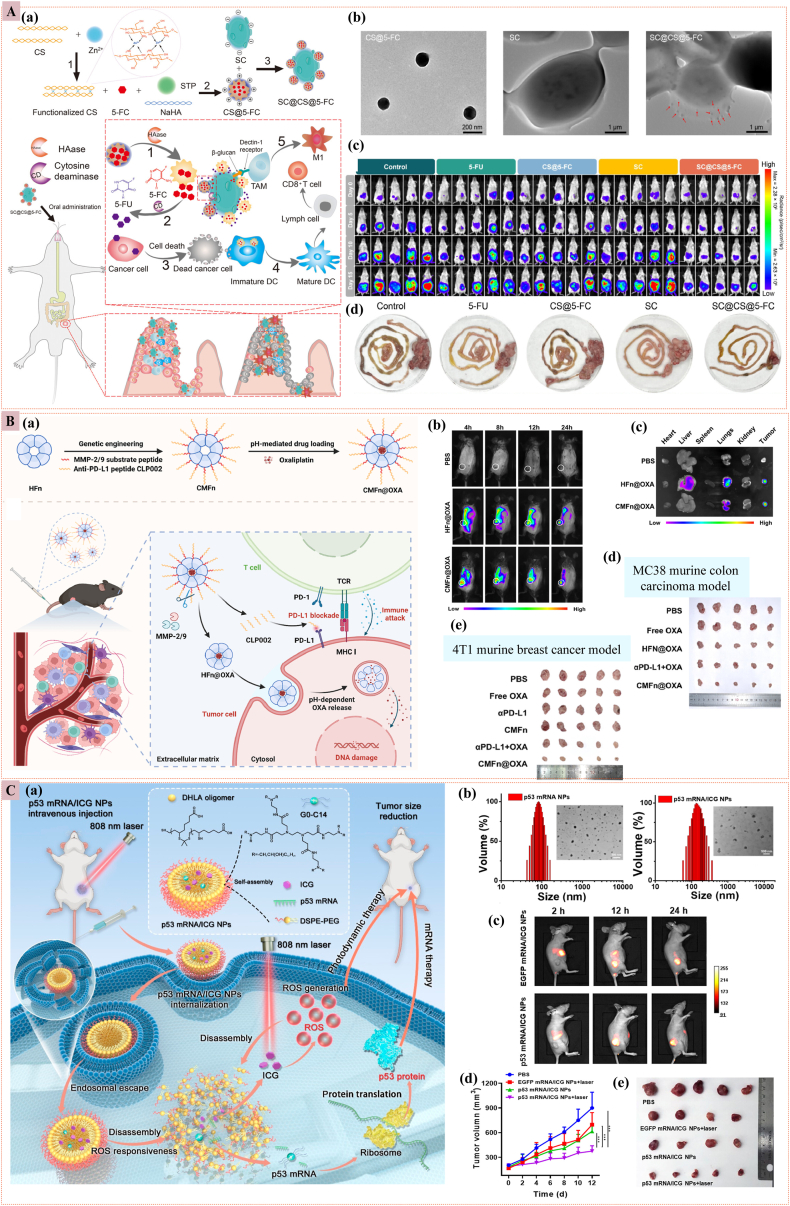
**(A)** Enzyme-responsive nanomedicine (a) Stepwise graphical overview of the *in-situ* chemotherapeutic nanogenerator SC@CS@5-FC (top) and its proposed oral administration route for colorectal cancer therapy (bottom). (b) TEM micrographs of CS@5-FC, SC, and SC@CS@5-FC nanoparticles (c) Longitudinal bioluminescence snapshots of tumor-bearing mice captured at predefined intervals after the indicated treatments. (d) Photographic record of excised tumors at study endpoint, illustrating morphological response to each regimen [65]. **(B)** (a) Conceptual diagram detailing the assembly of CMFn@OXA and its cascade anti-tumor mechanism following intravenous delivery. (b) *In vivo* whole-body fluorescence images and (c) *Ex vivo* organ scans acquired from MC38 tumor-bearing C57BL/6 mice 24 h after a single tail-vein injection of either Cy5.5-labeled HFn@OXA or Cy5.5-labeled CMFn@OXA. (d) Representative photographs of resected tumors from the MC38 murine colorectal carcinoma model following the indicated therapeutic regimens. (e) Corresponding tumor images harvested from the 4T1 murine breast cancer model after the specified treatments [66]. **(C)** ROS-responsive nanomedicine (a) Schematic of p53 mRNA/ICG NPs for combined therapy. (b) DLS and TEM validate NP assembly before and after ICG loading. (c) *In vivo* NIR imaging tracks the accumulation of NPs in H1299 tumors over time. (e) Tumor growth curves mirror the tumor's imaging results. (f) Corresponding excised tumors at day 12 visually confirm treatment efficacy [69].

Enzymes orchestrate indispensable physiological processes throughout healthy tissues. In contrast, the TME exhibits markedly dysregulated enzymatic activity, with matrix metalloproteinases (MMP) (such as MMP-2 and MMP-9), cathepsins (lysosomal proteases), phospholipases, and oxidoreductases frequently overexpressed to meet malignant cells' heightened metabolic and proliferative demands [42,63]. Exploiting this enzymatic fingerprint, enzyme-responsive nanocarriers are engineered to liberate therapeutic payloads exclusively within neoplastic lesions, maximizing on-target potency while sparing healthy tissues. Recently, *Sharma* et al. prepared HA-UA/PTX nanoparticles rationally engineered by exploiting the intrinsic biology of triple-negative breast cancer (TNBC). CD44, a hyaluronan receptor overexpressed on TNBC cells, was targeted via a hyaluronic acid (HA) shell, while the tumor-enriched hyaluronidase (HAase) provided an enzyme-responsive “switch” for site-specific release. Covalent grafting of the hydrophobic triterpenoid ursolic acid (UA) to HA created an amphiphilic prodrug that self-assembles into 173 nm spherical micelles with a dense hydrophobic core capable of solubilizing paclitaxel (PTX; 77 % entrapment). The HA shell ensures colloidal stability under systemic dilution. In contrast, HAase-mediated cleavage of the HA backbone in the acidic endo-lysosomal milieu accelerates PTX release (∼78 % in 72 h). It liberates UA, thereby achieving the synchronized delivery of two mechanistically complementary agents. Cellular studies confirmed CD44-dependent uptake (blocked by free HA), demonstrated synergistic cytotoxicity, and enhanced mitochondrial apoptosis in MDA-MB-231 and 4T1 cells. *In vivo*, the nanocarrier prolonged systemic exposure, selectively accumulated in orthotopic 4T1 tumors, and induced 90 % tumor regression without significant weight loss, hemolysis, or organ toxicity. The design thus integrates active targeting, enzyme-triggered release, and carrier-drug synergy into a single, clinically translatable nanoplatform for TNBC combination therapy [64]. *Qin* et al. proposed an innovative oral enzyme prodrug therapy combined with immunotherapy for treating orthotopic colorectal cancer using a fungi-triggered *in situ* chemotherapeutics generator named SC@CS@5-FC. This system utilizes *Saccharomyces cerevisiae* (SC) to deliver a prodrug, 5-fluorocytosine (5-FC), to the tumor site. Upon reaching the tumor, the chemotherapeutic generator releases 5-FC in response to hyaluronidase in the TME, which is then converted to the toxic chemotherapy drug 5-fluorouracil (5-FU) by cytosine deaminase (CD) expressed by SC. These SC and zinc-coordinated CS nanoparticles also serve as immune adjuvants, activating antigen-presenting cells and thereby enhancing the therapeutic effect. The use of CS nanoparticles (CS@5-FC) in this system represents a key application of nanomedicine, enabling controlled drug release and immune activation. *In vitro* and *in vivo* experiments demonstrated that SC@CS@5-FC effectively inhibits tumor growth, activates immune cells, and prolongs the survival of mice in an orthotopic colorectal cancer model. This study provides a promising strategy for oral enzyme prodrug therapy in cancer treatment, addressing challenges in sustained enzyme expression and optimal biological distribution through the integration of nanotechnology (Fig. 5A) [65]. *Liu* et al. developed a dual-responsive engineered nanoparticle, CMFn@OXA, based on ferritin for enhanced tumor-targeted delivery and controlled release of an anti-PD-L1 peptide (CLP002) and the chemotherapeutic drug oxaliplatin (OXA). The study leveraged genetic engineering to modify the surface of human heavy-chain ferritin (HFn) with CLP002 linked by an MMP-2/9 responsive sequence, forming CMFn. This design enabled the controlled release of CLP002 in the TME through enzymatic cleavage by MMP-2/9, thereby blocking the PD-1/PD-L1 interaction. OXA was encapsulated within CMFn via pH-mediated disassembly/reassembly, ensuring its release in the acidic lysosomal environment of tumor cells. *In vitro* and *in vivo* experiments demonstrated that CMFn@OXA effectively accumulated in tumors, exhibited pH-dependent drug release, and significantly inhibited tumor growth while promoting anti-tumor immune responses. The enzyme-responsive mechanism, combined with the dual functionality of CMFn@OXA, showed superior efficacy compared to traditional therapies, highlighting its potential as a novel strategy for cancer immunotherapy (Fig. 5B) [66]. Despite the promising examples of enzyme-responsive nanomedicine, it is crucial to acknowledge the significant heterogeneity in enzyme expression (e.g., MMP, cathepsins) across different tumor types and within the same tumor. For instance, while some tumors may overexpress Cathepsin B, others Cathepsin L, and many express both MMP-2/9 and various cathepsins to varying degrees [67,68]. To overcome this fundamental barrier, therapeutic strategies must move beyond one-size-fits-all designs and adopt personalized approaches, tailoring nanocarriers to the unique enzymatic signature of a patient's tumor to ensure robust therapeutic activation.

##### 3.2.1.4 ROS-responsive nanoparticles

Solid tumors sustain an aberrant redox landscape characterized by overexpressed superoxide dismutase and markedly elevated intracellular ROS, providing a tumor-exclusive cue for stimulus-responsive nanomedicine. H_2_O_2_, the most abundant and stable ROS species in malignant cells, serves as both a chemical trigger and a substrate for therapeutic activation. ROS-responsive nanocarriers, engineered from thioethers, acrylate amines, oxalates, thioacetals, or selenium/tellurium scaffolds, undergo rapid hydrophilic transition upon ROS encounter, enabling on-demand payload liberation. Despite excellent biocompatibility, thioether-based systems are susceptible to premature oxidation in specific microenvironments, necessitating molecular tuning to preserve activation quality [41,70]^.^ Metal-containing nanoparticles (Au, Cu, Mn) further amplify ROS utilization by catalyzing the decomposition of H_2_O_2_ into cytotoxic •OH via Fenton or Fenton-like chemistry. These catalytic nanoreactors remain inert in healthy tissues but undergo physicochemical transformation within TME, ensuring site-specific activation [[71], [72], [73]]. *Wu* et al. exemplified this paradigm with Fe(III)-WS_2_-polyvinylpyrrolidone nanocapsules that simultaneously load DOX and generate •OH, enabling multimodal chemodynamic, photothermal, and photodynamic eradication of cancer cells with minimal systemic exposure [74]. Beyond direct cytotoxicity, ROS-responsive platforms modulate tumor hypoxia by converting H_2_O_2_ to O_2_, thereby potentiating oxygen-dependent photodynamic therapy (PDT). Ferrocene derivatives, leveraging endogenous H_2_O_2_ for Fenton-driven •OH production, enhance chemodynamic therapy (CDT) selectivity while circumventing drug-resistance pathways [75,76]. Collectively, ROS-triggered nanomedicines exploit the tumor's oxidative imbalance to direct precise, multi-mechanistic cell death, positioning them as powerful tools in personalized oncology. A recent innovative approach is the development of ROS-responsive nanoparticles (SN38-CA@FC NPs), which combine chemotherapy and ferroptosis for enhanced anti-tumor efficacy, demonstrating targeted drug release. These nanoparticles were designed with a thioacetal linker that connects the potent chemotherapeutic agent SN38 to cinnamic aldehyde (CA), forming a dimeric prodrug. The prodrug was co-assembled with ferrocene carboxaldehyde (FC) to create nanoparticles that release SN38 and CA in response to ROS, thereby inducing DNA damage and promoting the Fenton reaction. This dual-action mechanism enhances the therapeutic effect and establishes a self-reinforcing feedback loop, where the released CA further amplifies ROS production. Both *in vitro* and *in vivo* studies demonstrated significant antitumor activity, highlighting the potential of ROS-responsive nanoparticles to improve cancer treatment outcomes by integrating chemotherapy with ferroptosis [77]. *Zhou* et al. developed a ROS-responsive polymeric nanoparticle platform for the codelivery of mRNA and a photosensitizer, integrating mRNA therapy with PDT for effective cancer treatment. The study focused on delivering p53 mRNA and indocyanine green (ICG) using ROS-responsive oligomer-based NPs. The NPs disassemble in response to ROS, enhancing mRNA translation efficiency and inducing apoptosis via p53 expression. Simultaneously, the released ICG generates ROS under 808 nm laser irradiation, inducing PDT. This combinatorial approach demonstrated significant anti-tumor effects in a lung cancer model, with efficient mRNA delivery and PDT, highlighting the potential of this strategy for treating cancers with p53 deficiency (Fig. 5C) [69]. However, the efficacy of ROS-responsive nanomedicine is constrained by a fundamental pharmacokinetic challenge: the marginal (∼100-fold) 'oxidation window' between basal blood H_2_O_2_ (≤5 μM) and elevated tumor concentrations (≥0.5 mM). This necessitates a compromise between systemic stability and intratumoral activation nanocarriers that resist premature cleavage in circulation often exhibit sluggish drug release. At the same time, those with rapid-release kinetics risk significant payload loss from leukocyte-derived oxidants [[78], [79], [80]]. Compounding this, profound intra-tumoral ROS heterogeneity within a single tumor underscores the need for real-time ROS sensors to personalize treatment, rather than employing a universal approach.

#### 3.2.2 Exogenous stimuli-responsive nanoparticles

##### 3.2.2.1 Light/photo-responsive nanoparticles

**Fig. 6:**
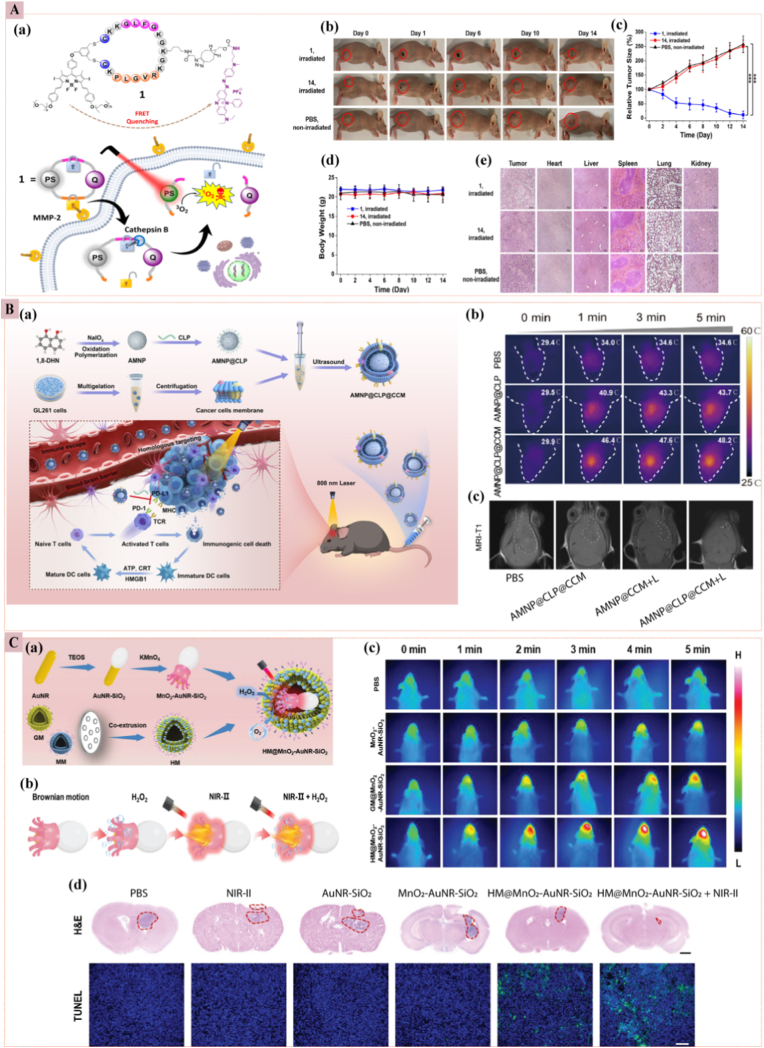
**(A)** Light/photo-responsive nanomedicine (a) Double-locked PMB 1: a tumor-selective photodynamic beacon requiring dual-enzyme co-activation. (b) *In vivo* photos of A549-tumor nude mice before/after intratumoral 1 or 14 + 680 nm laser (0.3 W cm^−2^, 180 J cm^−2^) or PBS, tracked for 14 days. (c) Tumor-growth curves mirror the visual responses. (d) Body-weight trends confirm negligible systemic toxicity. (e) H&E images of tumors and organs corroborate localized phototoxicity and safety [85]. **(B)** AMNP@CLP@CCM for GBM Photothermal-Immunotherapy (a) Stepwise assembly of AMNP@CLP@CCM and its cascade: magnetic guidance, then NIR-II photothermal ablation, next ICD-driven antitumor immunity. (b) *In vivo* IR thermography of orthotopic GBM mice after i.v. PBS, AMNP@CLP or AMNP@CLP@CCM; CCM coating prolongs tumor retention and raises peak ΔT. (c) Day-26 T_1_-weighted MRI confirms noticeable tumor volume reduction only in the AMNP@CLP@CCM group [86]. **(C)** HM@MnO_2_-AuNR-SiO_2_ nanomotor for GBM (a) One-pot synthesis schematic of HM@MnO_2_-AuNR-SiO_2_: hyaluronic-acid camouflage over MnO_2_-decorated gold nanorods within a mesoporous silica shell. (b) Propulsion diagram: endogenous H_2_O_2_ fuels MnO_2_ leading to O_2_ micro-bubbles, driving directional nanomotor movement toward hypoxic tumor cores. (c) IR thermal snapshots of GL261-luc orthotopic mice: highest intracranial temperature achieved with HM@MnO_2_-AuNR-SiO_2_ + NIR-II. (d) Brain-section histology: H&E (necrosis) and TUNEL (apoptosis) staining only show extensive tumor cell death in the nanomotor + NIR-II [87]. (For interpretation of the references to color in this figure legend, the reader is referred to the Web version of this article.)

Light-triggered drug release is a viable strategy for remote-controlled drug delivery [81]. This method destabilizes and breaks down photosensitive materials using specific wavelengths of light, thereby initiating the release of drugs. Ultraviolet (UV) light, sensitive to most photosensitive chromophores, is commonly used as a trigger. For instance, *Jiang* et al. synthesized an amphiphilic block copolymer of PEO and a hydrophobic poly(methacrylate) with an attached pyrene derivative. This construct releases Nile red upon UV light irradiation, following the cleavage of the pyrene groups [82]. However, UV light has limited tissue penetration due to absorption by other substances, posing a significant threat to healthy cells. This poor bioavailability restricts its application in nanomedicine-based drug delivery systems. In contrast, NIR light, with wavelengths ranging from 780 to 1700 nm, penetrates tissues more deeply and causes less cellular damage [41]. Consequently, researchers are increasingly focusing on developing NIR light-responsive nanomedicines for safer and more efficient drug delivery. For instance, micelle-based nanoparticles, which have hydrophobic cores and incorporate light-sensitive chromophore structures, can efficiently deliver drugs. Upon exposure to light, these micelles dissociate due to changes in the balance between hydrophilic and hydrophobic properties. *Tong* et al. investigated the PEG-PPyMA (poly(1-pyrenylmethyl methacrylate)) system, a type of light-sensitive micelle [83]. *Stenhouse* et al. developed a photo-responsive carrier based on conjugated polymer nanoparticles, such as poly(p-phenylene vinylene), which induces a photo-responsive effect leading to the swelling and opening of nanoparticles, thereby facilitating drug release [84]. The light exposure destabilizes the carrier by altering its molecular structure. NIR light-responsive systems operate through three main mechanisms: (1) Photoreactions in chromophores, initiated by two-photon absorption or up-conversion, result in bond breakages and conformational changes, leading to nanoparticles destabilization and drug release; (2) Photooxidation, involving the interaction of ROS generated by NIR light with photosensitizers, causes hydrophobic-hydrophilic transitions and subsequent polymer degradation, facilitating drug release; and (3) Photo-thermal effects, initiated by photothermal conversion agents, generate heat that leads to the disintegration of nanomedicines. The by-products of these reactions must be non-toxic and biodegradable [41]. In a significant advancement for targeted PDT in cancer treatment, researchers have developed a novel photodynamic molecular beacon (PMB) that functions as a biological AND logic gate, activated specifically by two tumor-associated enzymes. The PMB, designated as compound 1, integrates a distyryl boron dipyrromethene (DSBDP)-based photosensitizer with a Black Hole Quencher 3 (BHQ-3) moiety, connected via two peptide segments that serve as substrates for matrix metalloproteinase-2 (MMP-2) and cathepsin B, enzymes commonly overexpressed in cancer cells. Due to efficient Förster resonance energy transfer (FRET), the PMB is quenched in its native form. Still, it becomes activated upon cleavage by both enzymes, thereby restoring the photodynamic activity of the DSBDP unit. This dual-enzyme-responsive design enhances tumor specificity and minimizes side effects such as skin photosensitivity. Extensive *in vitro* and *in vivo* studies demonstrated significant tumor suppression in A549 tumor-bearing mice without notable toxicity, underscoring the potential of this PMB for precise PDT (Fig. 6A) [85]. *Sun* et al. developed a biomimetic nanotherapeutic platform (AMNP@CLP@CCM) for targeted PTT and immune checkpoint blockade (ICB) in orthotopic GBM. The platform utilizes allomelanin nanoparticles (AMNPs) as photothermal agents and carriers for the low-molecular-weight PD-L1 inhibitor CLP002. The nanoparticles are coated with cancer cell membranes (CCM) to enhance their ability to cross the BBB and target GBM tissues. The study demonstrated that AMNP@CLP@CCM can effectively cross the BBB, deliver CLP002 to GBM tissues, and induce photothermal ablation of tumor cells under 808 nm laser irradiation. The PTT enhances BBB penetration and upregulates PD-L1 expression on GBM cells, promoting T lymphocyte infiltration and amplifying the anti-tumor immune response to ICB therapy. *In vivo* experiments in an orthotopic GBM mouse model showed significant inhibition of tumor growth and prolonged survival, highlighting the potential of this nanoplatform for GBM treatment (Fig. 6B) [86]. To overcome the immunosuppressive environment of GBM and the BBB, researchers developed a biomimetic hybrid cell membrane-modified dual-driven heterojunction nanomotor (HM@MnO_2_-AuNR-SiO_2_). NIR-II (NIR-II) light and oxygen bubbles generate dual-driven propulsion in the MnO_2_-AuNR-SiO_2_ combination, enabling effective therapy at deep tumor sites. Electron-hole pairs dissociate and produce ROS, which kill immunogenic tumor cells under NIR-II laser irradiation. Additionally, MnO_2_ in the TME releases Mn^2+^ ions, triggering the cGAS-STING pathway and enhancing antitumor defense (Fig. 6C) [87]. This tailored approach enhances treatment precision, reduces adverse effects, and allows personalized dosing. However, light/photo-responsive nanomedicine continues to face significant challenges, including limited tissue penetration, off-target phototoxicity, oxygen dependence, uncertain long-term biocompatibility, and the absence of standardized dosimetry protocols [88,89]. These limitations underscore the need for clinically translatable photoabsorbers, optimized light-delivery systems, and rigorous safety evaluation frameworks to enable reliable and widespread clinical implementation.

##### 3.2.2.2 Temperature-responsive nanoparticles

**Fig. 7:**
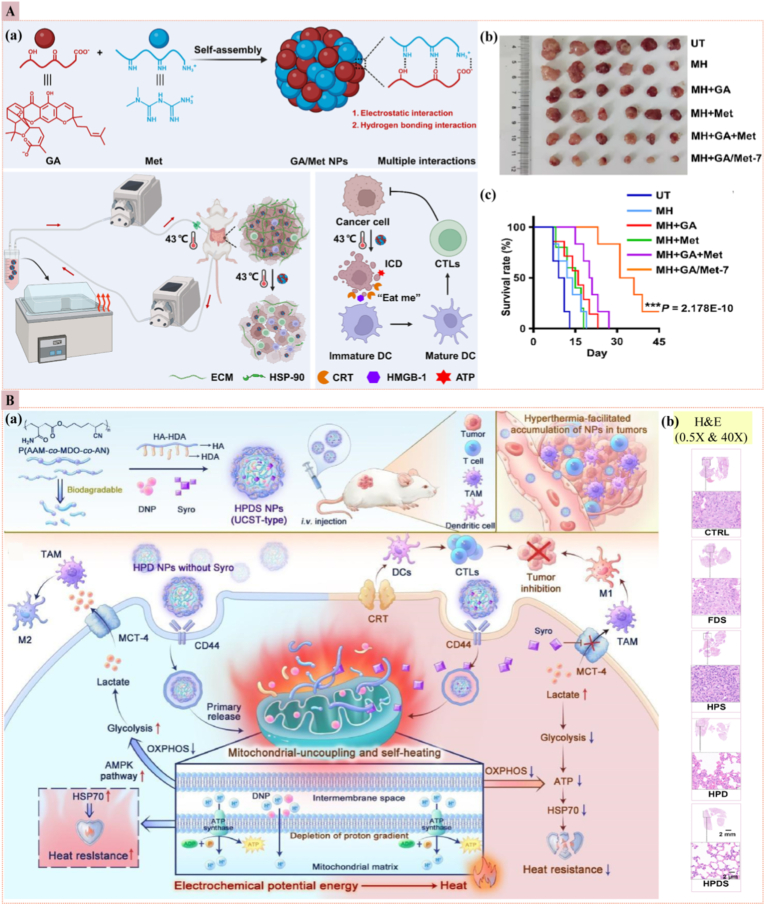
**(A)** Temperature-responsive nanomedicine (a) GA/Met NPs self-assemble via electrostatic and hydrogen bonds, co-load gambogic acid (GA) and metformin (Met), and were delivered by HIPEC to peritoneal tumors. Upon mild hyperthermia (MH) (43 °C), the nanoparticles (GA/Met NPs) trigger immunogenic cell death (ICD), thereby amplifying dendritic cell maturation and cytotoxic T-cell infiltration. (b) Representative excised tumors at day 9: the smallest masses in the MH + GA/Met-7 group (c) Kaplan–Meier survival of orthotopic CT26 mice: the longest median survival in MH + GA/Met-7 [94]. **(B)** UCST (upper critical solution temperature) nanomedicine (a) One-pot assembly of UCST-type HPDS NPs (HA-HDA + P(AAM-co-MDO-co-AN)), i.v. delivery, tumor accumulation via HA-CD44 binding, MH-triggered UCST collapse, mitochondrial uncoupling (lactate↓, OXPHOS↑), ICD amplification, and M1-TAM (anti-tumor immunity) polarization. (b) H&E of lung metastases: markedly fewer nodules after HPDS NPs treatment [95].

The inherent temperature difference between normal tissues, which maintain a temperature around 37 °C, and tumor tissues, which often exhibit temperatures ranging from 40 to 42 °C [90], has positioned temperature-responsive nanoparticles as a promising avenue for precise and controlled drug delivery in cancer treatment. These nanoparticles are designed to preserve their payload at ambient temperatures and release the drug only upon exposure to the elevated temperatures characteristic of TME. This targeted release mechanism minimizes systemic exposure and enhances therapeutic efficacy. Temperature-responsive nanoparticles comprise various materials, including polymeric substances, organic nanogels, and inorganic compounds. Among these, poly(N-isopropyl acrylamide) (PNIPAM) stands out as a widely studied temperature-responsive polymer due to its low critical solution temperature (LCST) of approximately 32 °C [91]. At this temperature, PNIPAM transitions from a hydrophilic to a hydrophobic state, facilitating the release of encapsulated drugs. The LCST of PNIPAM can be fine-tuned by copolymerizing it with other monomers, such as N, N-dimethyl acrylamide [92]. This modification enhances the hydrophobic interactions and improves the efficacy of temperature-sensitive carriers for targeted drug delivery. By leveraging the mild hyperthermia of tumor tissues, temperature-responsive nanoparticles achieve site-specific drug release with reduced systemic toxicity. This targeted approach enhances the therapeutic index of the delivered drugs and improves patient outcomes by reducing adverse effects associated with conventional chemotherapy. *Chen* et al. engineered thermo-responsive composite nanoparticles (CNPs) using hydroxybutyl CS oligosaccharide (HBCOS) and sodium caseinate (SC) for DOX-targeted cancer therapy. These CNPs, created via electrostatic interactions and covalent crosslinking, exhibited temperature-responsive behavior due to hydrogen bond breakage and chain shrinkage. The CNPs demonstrated concentration-independent thermo-sensitivity, stability, and biocompatibility, with higher DOX release rates at 42 °C compared to 37 °C, especially at lower pH levels. *In vitro* studies confirmed their temperature-responsive anti-tumor activity and cellular uptake, highlighting their potential as effective nanocarriers for controlled drug delivery in cancer treatment [93]. *Huapan Fang* et al. developed a novel carrier-free multifunctional nanomedicine platform using gambogic acid (GA) and metformin (Met) for enhanced hyperthermic intraperitoneal chemotherapy (HIPEC) against abdominal pelvic tumors. The GA/Met nanoparticles (GA/Met NPs) were self-assembled through multiple interactions, including electrostatic and hydrogen bonding. They were designed to improve the efficacy of HIPEC for treating orthotopic colorectal and ovarian cancers. Mild heat (MH) used in HIPEC induced ICD in cancer cells, releasing damage-associated molecular patterns (DAMPs) that enhanced the immunogenicity of the TME. GA acted as both a chemotherapeutic agent and an inhibitor of heat shock protein 90 (HSP-90), increasing the sensitivity of cancer cells to hyperthermia-induced cell death. Met cleared the tumor ECM, facilitating deeper penetration of nanoparticles into tumor tissues and promoting the infiltration of cytotoxic T lymphocytes. The synergistic combination of GA and Met during HIPEC triggered a robust anti-tumor immune response *in vivo*. In orthotopic colorectal and ovarian cancer models, GA/Met NPs significantly suppressed tumor growth and extended the survival of tumor-bearing mice, with no notable toxicity. This integrated strategy offered a promising therapeutic avenue for managing advanced abdominal pelvic tumors, with potential for broad clinical applications (Fig. 7A) [94]. *Zhe Yang* et al. developed a CD44-targeted, thermal-responsive nanocarrier for the co-delivery of 2,4-dinitrophenol (DNP) and syrosingopine (Syro) to induce endogenous hyperthermia and regulate immunometabolism in cancer cells. The objective was to enhance anti-tumor immune responses by leveraging the synergistic effects of endogenous hyperthermia and immunometabolism regulation. DNP, a mitochondrial uncoupler, converts the electrochemical potential energy of the inner mitochondrial membrane into heat, facilitating endogenous hyperthermia and inducing ICD in tumor cells. Syro inhibits excessive lactate efflux caused by DNP, downregulates tumor cell glycolysis, and alleviates immunosuppression and heat shock protein (HSP)-dependent thermo-resistance. The combined action of DNP and Syro impairs oxidative phosphorylation (OXPHOS) and glycolysis, leading to ATP depletion and tumor starvation. This strategy enhances tumor immunogenicity and reshapes the tumor immune microenvironment, effectively suppressing the growth of subcutaneous tumors and patient-derived organoids in TNBC. The study demonstrates that this endogenous hyperthermia strategy could revolutionize traditional hyperthermia for cancer treatment by eliminating the need for external equipment, broadening the applicability of hyperthermia in clinical settings (Fig. 7B) [95]. However, these nanoplatforms, locked to the narrow 40–42 °C window, therefore their selectivity is compromised by mild whole-body hyperthermia (∼39–40 °C) from fever, inflammation, or off-target energy deposition, triggering off-tumor activation [96] and fueling demand for closed-loop feedback instead of fixed thermal dosing to ensure precision.

##### 3.2.2.3 Magnetic field-responsive nanoparticles

**Fig. 8:**
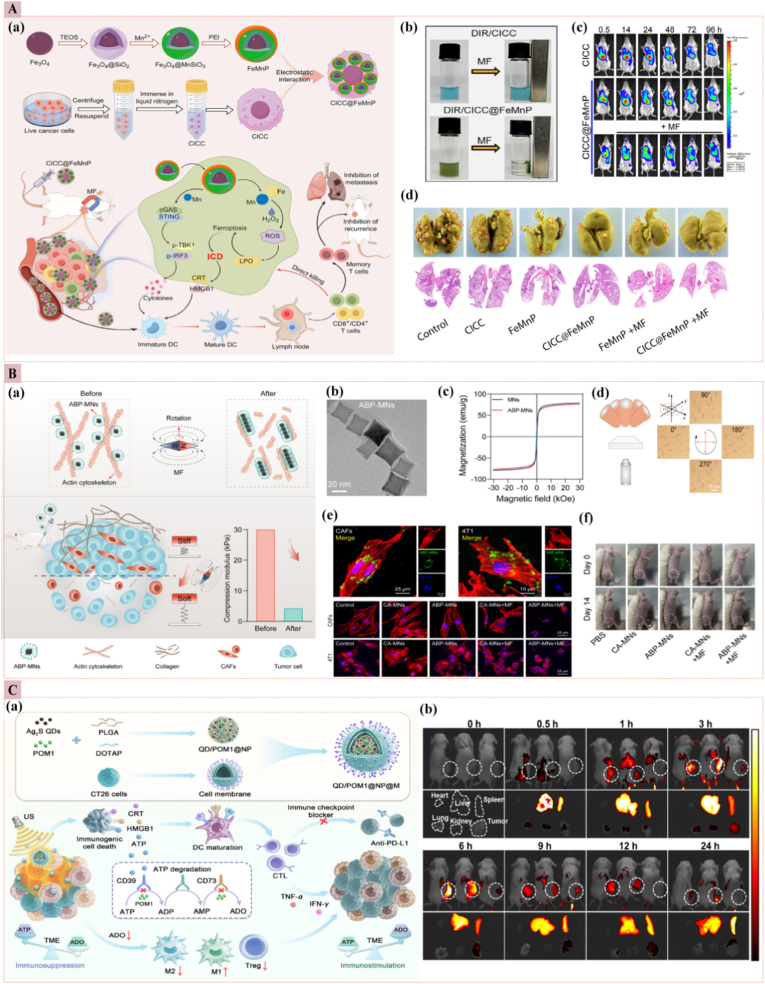
**(A)** Magnetic field-responsive nanomedicine (a) CICC@FeMnP biohybrid micromotors stepwise assembly & mechanism (b) CICC alone vs. CICC@FeMnP ± magnetic field (MF). (c) *In vivo* fluorescence images of tumor-bearing mice 24 h post-i.v. DIR-CICC or DIR-CICC@FeMnP, ± MF. (d) Lung metastasis: red-circled nodules and black-arrowed H&E lesions are smallest/absent after CICC@FeMnP + MF [99]. **(B)** (a) ABP-MNs: magnetically driven nanospinners that mechanically remodel actin in CAFs/4T1 cells, softening the tumor mechanical microenvironment (TMME). (b) TEM: uniform ABP-MNs. (c) Hysteresis: superparamagnetic MNs remain after surface conjugation. (d) Rotation assay: 5 mT/1 Hz MF keeps ABP-MNs spinning stably in solution. (e) Confocal images, where ABP-MNs colocalize with actin; 15 Hz/30 min MF collapses F-actin in CAFs and 4T1 cells, unlike CA-MNs. (f) MDA-MB-231 tumors shrink markedly by day 14 only after ABP-MNs + MF, linking cytoskeletal disruption to *in vivo* efficacy [102]. **(C)** USresponsive nanomedicine (a) Assembly of QD/POM1@NP@M: quantum dots + POM1 (CD39 inhibitor) wrapped in homotypic membrane. After i.v. injection, the membrane targets tumors; ultrasound triggers ICD (CRT/HMGB1/ATP release), DC maturation, and CTL activation. Simultaneously, POM1 blocks CD39/CD73/ADO, shifting TAMs from M2 to M1 and suppressing Tregs, thereby amplifying antitumor immunity. (b) *In vivo* fluorescence imaging of CT26 mice: substantial tumor accumulation of QD/POM1@NP@M at 6–24 h, with minimal off-target signal [105]. (For interpretation of the references to color in this figure legend, the reader is referred to the Web version of this article.)

Magnetic field-responsive nanoparticles have emerged as a promising strategy for enhancing the bioavailability, specificity, and safety of cancer therapeutics. These nanoparticles leverage external magnetic fields to direct their accumulation at tumor sites, thereby improving drug delivery efficiency and minimizing off-target effects [97]. MagForce Nanotechnologies, based in Berlin, Germany, has developed a magnetic hyperthermia system named NanoTherm®. This system employs an aqueous suspension of SPION with an iron concentration of 112 mg/mL. These biocompatible nanoparticles are administered directly to the tumor site and subjected to an alternating magnetic field (AMF), resulting in localized heating that facilitates the ablation of tumor cells [98]. Recently, *Qingfei Zhang* et al. developed a novel therapeutic platform using cryo-inactivated cancer cells (CICC) derived magnetic micromotors (CICC@FeMnP) for tumor synergistic immunotherapy (Fig. 8A) [99]. These micromotors were engineered to target and accumulate at the tumor site under magnetic control. The FeMnP component induces ferroptosis, triggering ICD in tumor cells. Meanwhile, the CICC component, rich in tumor antigens, enhances the vaccination effect. Additionally, Mn^2+^ ions released from FeMnP activate the cGAS-STING pathway, stimulating the immune response. This multifunctional system facilitates the establishment of a collaborative anti-tumor immune network, and inducing long-lasting immune memory effects. In an orthotopic breast tumor mouse model, CICC@FeMnP significantly inhibited tumor progression, recurrence, and lung metastasis, thereby prolonging the overall survival of tumor-bearing mice. This study highlights the potential of engineered biohybrid micromotors as an innovative strategy in cancer immunotherapy, offering a promising alternative to enhance the efficacy of tumor treatment. *Tong* et al. demonstrated that AMF can heat magnetic Fe_3_O_4_ nanoparticles within tumor tissues with minimal adverse effects [100]. This approach leverages the unique characteristics of tumors, such as inadequate lymphatic drainage and rapid blood vessel development, to enhance drug delivery. Hyperthermia, which increases the temperature of paramagnetic or ferromagnetic materials, is an effective treatment modality for cancer. The heat generation of magnetic field-mediated nanoparticles depends on their magnetic relaxation rate and saturation magnetization, with maghemite (γ-Fe_2_O_3_) and Fe_2_O_3_ nanoparticles showing notable efficacy. Surface modifications with polymers such as PEG and CS reduce aggregation, enhance biocompatibility, and extend circulation times, thereby improving the therapeutic effectiveness of these nanoparticles. These modifications also enable precise drug delivery, ensuring targeted and controlled release at the tumor site [101]. Innovatively, *Xing Fan* et al. [102] introduced an actin-binding protein-modified magnetic nanomotor (ABP-MN) system, which, when coupled with a rotating magnetic field (MF), mechanically modulates the tumor mechanical microenvironment (TMME). The ABP-MNs, with their ultrasmall diameter of 23 nm, specifically target the actin cytoskeleton within cancer-associated fibroblasts (CAFs) and tumor cells. By inducing depolymerization of filamentous actin (F-actin) via magneto-mechanical force, these nanomotors reduce tumor matrix stiffness, enhance immune cell infiltration, and inhibit tumor growth. In preclinical studies using 4T1 tumor-bearing mice, ABP-MNs combined with MF achieved a remarkable 95.8 % tumor growth inhibition and significantly extended survival rates. This study underscores the potential of intracellular mechanical modulation of the TMME via ABP-MNs as a novel and effective mechano-based therapy for solid tumors (Fig. 8B). Although recent advances are encouraging, magnetic field–responsive nanomedicine still faces critical limitations related to field penetration, thermal control, nanoparticles stability, biodistribution, and regulatory standardization. Overcoming these barriers demands harmonized dosimetry, scalable fabrication, and comprehensive *in vivo* evaluation to achieve clinical applicability [103,104].

##### 3.2.2.4 US-responsive nanoparticles

The US plays a pivotal role in cancer treatment, leveraging high power to eliminate cells and low power for imaging and diagnosis. The essential criteria for US-mediated exogenous stimuli-responsive nanoparticles include responsiveness to US power, stable drug encapsulation, and imaging-controlled delivery [106]. US waves enhance drug penetration through mechanical and thermal mechanisms, offering high biocompatibility and efficient drug delivery. For instance, a targeted US-mediated therapy involves nanoparticles linked by oxyl-alkyl hydroxylamine bonds, connecting a hydrophobic stearic segment to a hydrophilic pullulan. Upon exposure to US, these bonds break, triggering the release of therapeutic drugs directly at the tumor site, effectively suppressing cancer growth with precise and timely delivery. The US not only triggers drug release but also enhances drug penetration by creating membrane pores, facilitating deeper tissue delivery. These nanoparticles are often decorated with targeting ligands, enabling active and passive targeting for more precise and effective therapy [107]. The integration of sonodynamic treatment (SDT), which utilizes US to generate toxic singlet oxygen (^1^O_2_), with immune agents such as programmed death-ligand 1 (PD-L1) and programmed death-1 (PD-1), demonstrates potential in cancer treatment. Challenges with organic sono-sensitizers are overcome by using nanomedicine like mesoporous silica, which enhances treatment efficacy. Multifunctional US-responsive nanoparticles further optimize tumor treatment by combining chemotherapy and SDT, enabling targeted cell eradication and activation of the immune response. *Yuanyuan Zhang* et al. developed a metabolic reprogramming nanomedicine (QD/POM1@NP@M) that enhances colon cancer sonodynamic immunotherapy by inhibiting the CD39/CD73/ADO pathway. This nanomedicine encapsulates sonosensitizers (Ag_2_S quantum dots) and the CD39 inhibitor POM1, and is coated with homologous tumor cell membranes to enhance its targeting capabilities. The multifunctional nanocarrier induces ICD through SDT, releasing ATP and other damage-associated molecular patterns (DAMPs) that activate immune responses. Simultaneously, POM1 inhibits CD39 activity, reducing the conversion of ATP to immunosuppressive adenosine (ADO), thereby mitigating the immunosuppressive TME. *In vivo* studies demonstrated significant anti-tumor efficacy, with QD/POM1@NP@M facilitating the infiltration of anti-tumor immune cells and reducing the number of immunosuppressive cells. Combined with the checkpoint inhibitor α-PDL1, the nanomedicine enhanced systemic anti-tumor immunity and promoted long-term immune memory. This study presents a novel approach for combining non-invasive SDT and ATP-driven immunotherapy, providing new insights for future cancer treatments (Fig. 8C) [105]. Although US-responsive nanomedicine enables non-invasive, spatiotemporally controlled drug delivery, several limitations impede clinical translation. Achieving precise localization without off-target heating or cavitation-induced damage remains challenging, and variable acoustic thresholds across tissues lead to inconsistent drug release. Tumor heterogeneity and limited cavitation in deep or hypoperfused regions further reduce efficacy. The lack of standardized acoustic parameters, uncertain long-term biocompatibility of gas-generating agents, and translational gaps between animal models and human anatomy continue to fuel debate over its clinical superiority to simpler stimulus-responsive systems [[108], [109], [110]].

##### 3.2.2.5 Radiation-responsive nanoparticles

**Fig. 9:**
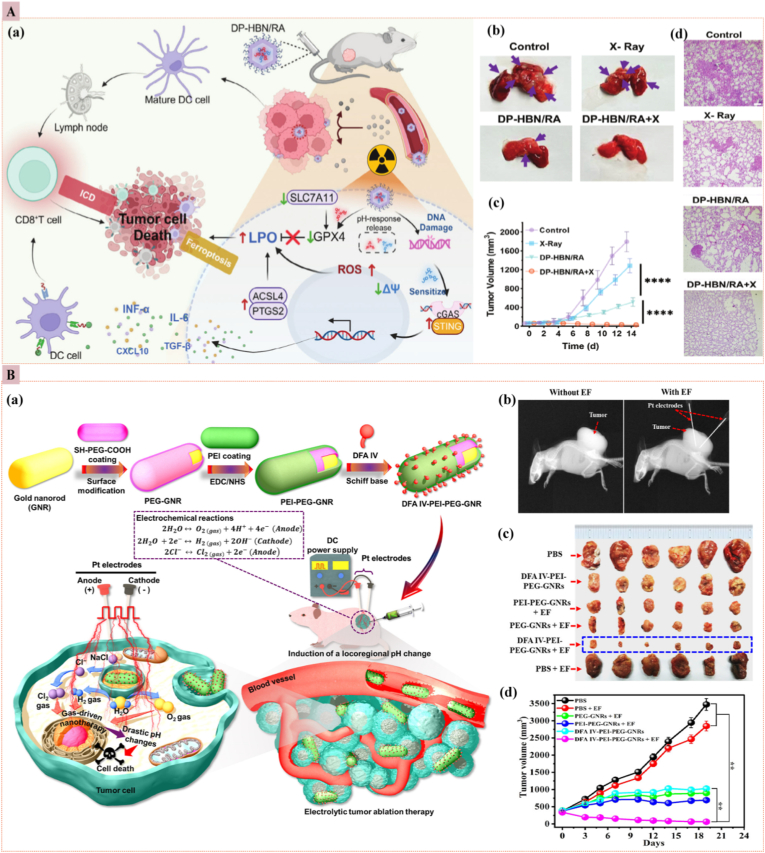
**(A)** Radiation-responsive nanomedicine (a) DP-HBN/RA with X-ray ignites ROS burst + GPX4 blockade, amplified ferroptosis, and immune system activation. (b) Lung images (day 14): DP-HBN/RA + X-ray shows minimal metastases (c) 4T1 tumor growth: DP-HBN/RA + X-ray yields sustained regression (d) H&E lung sections: scarce metastatic foci after DP-HBN/RA + X-ray [117]. **(B)** Electric field-responsive nanomedicine. (a) DFA IV-PEI-PEG-GNRs: seed-grown gold nanorods (GNRs), then PEG/PEI coating and DFA IV Schiff-base graft. Under DC, the nano-electrocatalyst splits water (O_2_, H_2_, Cl_2_), steeply acidifies the tumor milieu, and triggers lethal oxidative stress. (b) X-ray: CT-26 tumors after intra-tumoral DFA IV-PEI-PEG-GNRs ± 30 min square-wave DC; electrodes visible only in EF (electric field) + group. (c) Day-28 tumor photos: smallest/white necrotic masses in EF + cohort. (d) Tumor-volume curves: EF + group shows near-complete regression [122]. (For interpretation of the references to color in this figure legend, the reader is referred to the Web version of this article.)

Radiation therapy (RT), using gamma rays and X-rays, is a common cancer treatment but can cause side effects like radio-dermatitis. To address this, researchers are developing X-ray-activated nanoparticles that function efficiently at lower doses, reducing side effects while maintaining efficacy [111,112]. These nanoparticles release drugs upon X-ray exposure, producing ^1^O_2_ to enhance therapy [113]. Designs such as bionic nano-capsules or diselenide (-Se-Se-) nanocarriers convert X-rays into UV radiation, thereby improving drug release and reducing radiation exposure [114]. *Chen* et al. outlined X-ray-mediated cancer-targeted nanosystems that effectively eliminated systemic tumors at low radiation doses [111]. These nanoparticles promote tumor necrosis and suppress growth through oxidative stress and hypoxia, with X-ray-activated scintillators generating ROS to damage DNA [115]. Heavy metals like tantalum (Ta) serve as radiosensitizers by generating Auger electrons and photoelectrons, enhancing the effectiveness of X-ray radiation in nanomedicine for treating solid tumors. The high penetration depth of X-rays enables targeted therapy with minimal side effects, as demonstrated in a xenograft model where nanoparticles reduced tumor volume without causing weight loss or mortality [116]. *Aishajiang* et al. developed a pH-responsive nanomedicine (DP-HBN/RA) for enhancing RT in TNBC by concurrently amplifying ferroptosis and immune system activation. This nanomedicine is composed of hollow Bi_2_Se_3_ nanoparticles loaded with RSL3 (a ferroptosis inducer) and diABZi (a STING agonist), modified with DSPE-PEOz. It efficiently concentrates X-ray radiation energy within tumors, generating ROS that induce lipid peroxidation and ferroptosis. The acidic TME triggers the release of RSL3 and diABZi, which inhibit GPX4 to enhance ferroptosis and activate the cGAS-STING pathway to evoke a systemic immune response. *In vitro* and *in vivo* experiments demonstrated that DP-HBN/RA significantly enhances RT efficacy by increasing DNA damage, promoting ferroptosis, and boosting immune activation, effectively suppressing tumor growth and metastasis. This study provides a novel strategy for overcoming radioresistance in TNBC by integrating ferroptosis and immune activation with RT (Fig. 9A) [117]. Radiation-responsive nanomedicine holds promise for enhancing radiotherapy; however, its translation into the clinic remains hindered by several key limitations. The activation of nanoparticles is highly dependent on radiation dose, energy spectrum, and tumor uptake, resulting in variable dose enhancements across studies. Tumor heterogeneity and hypoxia reduce the efficacy of radiosensitizers, while off-target nanoparticle deposition and secondary radiation raise concerns about the safety of these treatments. Moreover, the lack of standardized dosimetry for nanoparticle-mediated radiation effects, together with uncertainties regarding long-term clearance and biocompatibility, presents significant barriers [118,119]. Addressing these issues will require harmonized radiation-based nanoparticle protocols, precise imaging-guided distribution mapping, and rigorous *in vivo* validation before widespread clinical adoption.

##### 3.2.2.6 Electric field-responsive nanoparticles

Electric fields, an exogenous non-invasive stimulation method, have been clinically employed in oncology, notably in the Food and Drug Administration (FDA) approved treatment of GBM. This method is advantageous for drug delivery, as high-intensity electric fields can directly affect cellular membrane permeability [120]. Furthermore, conductive polymers, such as polyaniline, polypyrrole, and poly(3,4-ethylenedioxythiophene), as well as conductive materials like carbon nanotubes, metals, and graphene nanoparticles, are utilized to enhance the performance of these systems [121]. *Joe* et al. introduced electric field-responsive gold nanoantennas for the treatment of colorectal cancer. They developed difructose dianhydride IV-conjugated polyethylenimine-polyethylene glycol-modified gold nanorods (DFA IV-PEI−PEG-GNRs) as electric nanoantennas and nanoelectrocatalysts for electrolytic ablation (EA) therapy. These nanoantennas generate electrolytic products, such as hydrogen, oxygen, and chlorine gases, when triggered by square-wave direct current (DC) fields, inducing local and regional pH changes and causing cell death (Fig. 9B) [122]. The study demonstrated that DFA IV-PEI−PEG-GNRs significantly enhanced EA effectiveness in treating colorectal cancer both *in vitro* and *in vivo*, offering a promising strategy to improve tumor specificity and control. While electric field–responsive nanomedicine enables precise, on-demand drug release under external stimulation, its clinical translation remains limited by several challenges. Electric field attenuation in biological tissues restricts penetration depth, and non-specific stimulation can induce unintended off-target effects. High-intensity fields may also generate Joule heating and electrochemical reactions, raising safety concerns. Furthermore, the instability of conductive polymers, the absence of standardized stimulation parameters, and limited *in vivo* validation hinder reproducibility and regulatory progress [[123], [124], [125]]. Overcoming these barriers will require optimized material design, harmonized safety standards, and integration with clinically adaptable field delivery systems to realize the full therapeutic potential of electric field–responsive nanomedicine.

#### 3.2.3 Dual/multiple stimuli-responsive nanoparticles

To optimize nanomedicine as drug carriers, they must exhibit high drug loading capacity, ensure targeted delivery to pathological sites, prevent premature drug leakage, and facilitate efficient *in situ* drug release. To achieve these goals, "smart" multiple stimuli-responsive nanoparticles have been developed, which respond to both internal and external stimuli [66,126]. Dual or multiple stimuli-responsive nanoparticles, which react to combinations of two stimuli signals concurrently or in succession, improve drug release regulation. This can also facilitate a more accurate and effective drug delivery system, enhancing anti-tumor efficacy in both *in vitro* and *in vivo* models. Combinations of dual stimuli-responsive nanoparticles include pH/ROS, pH/GSH, photo/ROS, pH/US, pH/photon, etc. The combination of multiple stimuli synergizes, allowing these systems to outperform nanoparticles responsiveness to a single stimulus, particularly in the complex TME. Dual stimuli-responsive systems primarily function at specific locations or in distinct stages, sequentially activating the release of drugs [127,128]. *Yang* et al. established photo/ROS-responsive nanoparticles that enabled controlled drug release, incorporating a photosensitizer and a ROS-sensitive bis-(alkylthio)alkene linker, which facilitated on-demand drug release through light activation, thereby improving therapeutic efficacy [129]. Nanoparticles that respond to both US and GSH have also been identified as potential candidates for synergistic tumor immunotherapy, effectively addressing issues like insufficient tissue depth penetration [130]. Recently, *Darya* et al. engineered 87 nm sized superparamagnetic Fe_3_O_4_ nanoparticles surface-decorated with locust-bean-gum mannan to co-deliver the bacterial alkaloid prodigiosin (PG) (PG@M-MNPs). The mannan shell serves a dual purpose: lectin-mediated targeting of mannose receptors overexpressed on MCF-7 breast and HepG2 liver cancer cells, and enzyme-responsive shedding by lysosomal mannanase, which triggers a first-order, controlled release of PG (∼38 % within 60 min). This selectivity resulted in a 2.2-fold higher Anti-Cancer Selectivity Index (ASI) for breast cancer cells versus free PG, sparing normal NIH/3T3 fibroblasts. Moreover, PG-loaded mannan-MNPs were avidly internalized by CD206^+^ M2 tumor-associated macrophages; low-dose formulations re-polarized M2 toward the pro-inflammatory M1 phenotype (↓ ARG-1, ↑ IL-6), whereas high concentrations drove a complete M2 to M1 switch. The system integrates magnetic tumor targeting, cancer-cell cytotoxicity, and immunomodulation within a single nanoplatform, offering a rationally designed strategy for chemo-immunotherapy against breast and liver malignancies [131]. *Zou* et al. introduced a TME-responsive hybrid nanomedicine, ZnPP@FQOS, designed to enhance photodynamic-immunotherapy in fibroblast-rich tumors through the strategic remodeling of cancer-associated fibroblasts (CAFs) and the amplification of ROS. This innovative approach utilizes a dual-responsive release mechanism, where hydrophilic quercetin (Que) and hydrophobic zinc protoporphyrin (ZnPP) are encapsulated within organosilica micelles. Upon reaching the tumor site, Que is released first, driven by the weak acidity of the TME, to remodel CAFs and alleviate hypoxia. Subsequently, ZnPP is released in response to the high GSH levels in tumor cells, enabling PDT upon laser irradiation. This sequential release enhances the tumor penetration and retention of the nanomedicine, amplifying ROS generation through the downregulation of antioxidant markers, such as HO-1, and inducing apoptosis. *In vivo*, studies in fibroblast-rich KP tumor-bearing mice demonstrated significant tumor suppression and systemic immune activation, with the ZnPP@FQOS + Laser group showing a tumor inhibition rate of 92.9 %. Furthermore, combining ZnPP@FQOS with anti-PD-L1 immunotherapy resulted in a robust abscopal effect, enhancing the efficacy of both primary and distant tumor treatments. This multifaceted approach underscores the potential of CAFs remodeling and ROS amplification for improving cancer therapy outcomes, highlighting a promising strategy for developing next-generation nanomedicines [132]. *Li* et al. designed a novel photothermal-responsive multi-enzyme nanoprobe, MnO_2_R@FePDAc, to enhance ferroptosis in oral squamous cell carcinoma (OSCC) by amplifying ROS and depleting GSH. The nanoprobe encapsulates the ferroptosis inducer RSL3 within hollow mesoporous manganese dioxide (HM-MnO_2_) nanoparticles, which are surface-modified with iron-doped dopamine (Fe-PDA) and cRGD tumor-targeting peptides. Under NIR irradiation, the nanoprobe exhibits a photothermal conversion efficiency of 39.1 %, triggering structural degradation and rapid release of RSL3. The photothermal effect significantly enhances the catalytic activity of the nanoprobe, mimicking the functions of peroxidase (POD), oxidase (OXD), GSH peroxidase (GPx), and NADH oxidase (NOx). These activities generate substantial ROS and efficiently deplete intracellular GSH, creating an optimal TME for inducing "explosive" ferroptosis. *In vitro* and *in vivo* experiments demonstrated significant tumor growth inhibition and biocompatibility, highlighting the potential of MnO_2_R@FePDAc as a powerful tool for cancer therapy through the synergistic combination of photothermal therapy and ferroptosis [133]. Multi-stimuli-based nanoparticles achieve programmable drug release, yet each additional responsive element increases synthetic steps, amplifies batch-to-batch variance, and lowers colloidal stability, while stimulus cross-reactivity distorts release profiles. The paucity of *in vivo* performance data, absence of harmonized assays, and emerging immunotoxicity signals collectively impede regulatory qualification. Clinical translation, therefore, demands simplified architectures, reproducible synthetic routes, and cost-scalable manufacturing frameworks [126,134,135].

### 3.3 Bio-inspired/bio-responsive nanomedicine

Beyond the classical physicochemical stimuli (pH, redox potential, temperature), living systems supply a rich and yet largely under-exploited array of endogenous biological cues that can be incorporated into nanoscale drug-delivery architectures. Two emerging paradigms now dominate this frontier: (i) microbiome-modulated nanomedicine, which leverages the tumor gut microbiota as both a biocatalytic reactor and an immunological rheostat; and (ii) exosome-based nanocarriers, which repurpose evolutionarily optimized intercellular vesicles for precision therapeutic transport [[136], [137], [138], [139], [140]]. Together, these bio-inspired systems combine the precision of nanotechnology with the adaptability of living biology, creating therapies that can respond to a patient's own biological environment in real time [136,140,141].

#### 3.3.1 Microbiome-modulated nanomedicine

**Fig. 10:**
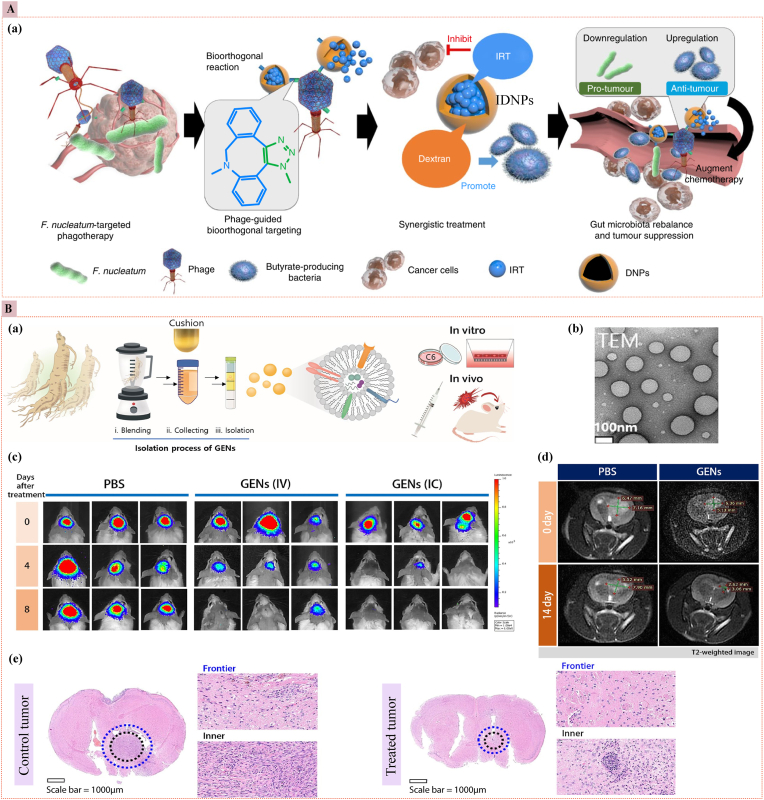
**(A)** Microbiome-modulated nanomedicine (a) Schematic illustration of phage-guided microbiome-modulating nanomedicine. Phages target colorectal cancer (CRC) and the pro-tumorigenic bacterium *F. nucleatum*, while bioorthogonally linked irinotecan-loaded dextran nanoparticles (IDNPs) inhibit these bacteria and enhance the anti-tumorigenic butyrate producers. Dextran enhances this effect by rebalancing the gut microbiota and augmenting chemotherapeutic efficacy [149]. **(B)** Exosome-based nanomedicine (a) Schematic representation of the isolation and characterization of ginseng-derived exosome-like nanoparticles (GENs) from fresh ginseng, Ginseng juice centrifugation, and subjected to sucrose gradient (68 % and 27 %) to isolate GENs. Followed density gradient purification (1.13–1.19 g/mL) and GENs' toxicity and efficacy *in vitro* and *in vivo.* (b) Round-shaped GENs under transmission electron microscopy (TEM). (c) Post orthotopic implantation, the luminescence of luciferase-expressing C6 cells was quantified using an *in vivo* imaging system. By day 8, the GEN-treated group exhibited a marked decrease in C6 glioma luminescence compared to controls. (d) MRI imaging of tumor volume after 14 days of treatment. (e) H&E staining revealed significant tumor size reduction in the treated group [155].

The gut and tumor microbiomes are now recognized as master regulators of oncogenesis, immune tone, and therapeutic efficacy, creating a paradigm shift toward microbiome-modulated nanomedicine [142]. This approach leverages the bidirectional interaction between nanocarriers and microbial communities to overcome treatment resistance and personalize oncology. The microbiome systemically shapes the TME. For instance, tumor-resident bacteria such as *Fusobacterium nucleatum* and *Escherichia coli* have been shown to promote colorectal carcinoma via modulation of immune pathways (e.g., NLRP3 inflammasome), thereby shaping the tumor TME toward immunosuppression and metastasis [143]. Microbial metabolites, including short-chain fatty acids (butyrate, propionate), secondary bile acids (deoxycholate, lithocholate), and inosine, remodel the TME by modulating immune checkpoint expression, altering stromal activation, and influencing cytotoxic T-cell function. These effects extend the classic "seed and soil" hypothesis of metastasis to include microbial contributions to the pre-metastatic niche [137,144,145]. Mechanistically, the microbiome interfaces with nanomedicine through two primary pathways. First, it acts as a bioreactor, where bacterial-specific enzymes (e.g., β-glucuronidase, azoreductase) can cleave prodrugs or nanocarrier components, enabling tumor-localized activation, particularly in colorectal and hepatic cancers. Second, it serves as an immune calibrator [138,144]. A dysbiotic microbiome promotes immunosuppression and inflammation, which can accelerate the clearance of nanocarriers via the mononuclear phagocyte system and diminish the efficacy of nano-immunotherapies. Conversely, a favorable microbiome enhances dendritic cell maturation and T-cell infiltration, synergizing with immunomodulatory nanocarriers. These insights are now being translated into therapeutic strategies. "Smart" nanoparticles are engineered with microbial enzyme-responsive linkers (e.g., azo-based polymers) for site-specific drug release in the gut or microbiome-rich tumors [[146], [147], [148]]. For instance, *Zheng* et al. introduced a sophisticated biotic–abiotic hybrid nanosystem to modulate the gut microbiota for enhanced colorectal cancer (CRC) therapy. Recognizing the pro-tumoral role of *Fusobacterium nucleatum* and the anti-tumoral function of butyrate-producing bacteria, the authors developed irinotecan-loaded dextran nanoparticles (IDNPs) that were covalently linked via bioorthogonal chemistry to azide-modified phages specific for *F. nucleatum*. This phage-guided system demonstrated precise targeting and elimination of the pro-tumoral bacteria within the TME, thereby reversing *F. nucleatum*-induced chemoresistance. Concurrently, the dextran nanoparticles promoted the proliferation of beneficial *Clostridium butyricum*, increasing the production of the anti-cancer metabolite butyrate. In both orthotopic and spontaneous CRC mouse models, this dual-action strategy, which selectively depletes a harmful species while enriching a beneficial one, significantly augmented the efficacy of first-line chemotherapy, offering a novel and precise approach to manipulating the TME via the gut microbiota (Fig. 10A) [149]. Furthermore, nanocarriers can be deployed to deliver microbiome-modulating agents, such as prebiotics, probiotic bacteria, or postbiotic metabolites, thereby reshaping the microbial landscape and reversing immunosuppression [150,151]. When co-administered with checkpoint inhibitor-loaded nanocarriers, these microbiome-targeting nano-adjuvants have shown synergistic anti-tumor effects by restoring immune responsiveness within the TME. Preclinical investigations further demonstrate that nanoparticles targeted toward tumor-associated microbes can effectively reprogram local immunity by promoting dendritic cell maturation (CD80^+^, CD86^+^ expression), enhancing cytotoxic CD8^+^ T-cell infiltration, and augmenting the therapeutic efficacy of checkpoint blockade [143,152,153]. Collectively, these findings highlight the potential of microbiome-responsive nanomedicine to convert immunologically “cold” tumors into “hot,” immune-active phenotypes, thereby overcoming one of the central barriers in cancer immunotherapy. Looking forward, integrating patient-specific microbial signatures into nanocarrier design represents a transformative step toward ecosystem-based precision oncology, extending the concept of personalization beyond genomic and proteomic profiling to include the unique microbial composition of each patient. However, key translational challenges remain, including the heterogeneity of tumor-resident microbiomes, potential off-target perturbation of commensal flora, the selection and biosafety validation of enzyme-responsive linkers, and the integration of microbiome profiling into clinical decision-making frameworks [136,154]. Addressing these issues will be essential for advancing microbiome-modulated nanomedicine from preclinical proof-of-concept to clinically viable, patient-tailored cancer therapeutics.

#### 3.3.2 Exosome-based nanomedicine

The paradigm of drug delivery in oncology is shifting towards biologically derived nanocarriers that offer superior biocompatibility and targeting specificity. Among these, exosomes, a subclass of extracellular vesicles (EVs), have emerged as front-runners [156]. These natural, cell-derived nanoparticles possess an innate ability to transport biomolecules between cells, evade immune clearance, cross biological barriers like the BBB, and have a natural tropism for specific tissues, making them exceptionally well-suited as drug carriers [157,158]. Native exosomes, particularly those derived from dendritic cells or mesenchymal stem cells (MSCs), inherently carry biomolecules and exhibit tumor-homing capabilities, the ability to cross the BBB, and immune evasion via CD47 “don't-eat-me” signaling. For instance, MSC-derived exosomes loaded with DOX through sonication demonstrated approximately 12-fold higher intra-tumoral accumulation while reducing cardiotoxicity in orthotopic breast cancer models, illustrating the therapeutic potential of unmodified exosomes [139,[159], [160], [161]]. Recently, *Kim* et al. established ginseng-derived exosome-like nanoparticles (GENs) as a potent anti-glioma platform, demonstrating their ability to cross the BBB and target tumor cells. The study revealed that GENs induce apoptosis through BAX/BCL-2 modulation and remodel the immunosuppressive TME by driving M2 to M1 macrophage repolarization and suppressing cancer-associated fibroblasts. A key mechanistic insight was the role of the plant miRNA ptc-miR396f in downregulating the c-MYC oncogene. This work underscores the significant potential of plant-derived exosomes as natural, multifunctional nanocarriers for targeted glioma therapy (Fig. 10B) [155]. Beyond native properties, engineered exosomes offer precision-targeted therapy. Surface functionalization through genetic engineering, such as Lamp2b fused with iRGD peptides, anti-HER2 single-chain variable fragments, or nanobodies, or chemical conjugation via click-chemistry, enables tumor-specific delivery [161,162]. Multi-cargo exosomes can simultaneously transport chemotherapeutics, siRNA, and pro-drugs, supporting integrated chemo–gene–immunotherapy strategies. Recent preclinical studies have highlighted these advances: hybrid exosomes formed by fusing MSC-derived vesicles with folate-functionalized liposomes and loaded with PTX significantly suppressed tumor growth in orthotopic models, decreased M2-type tumor-associated macrophages, and promoted M1 polarization. Similarly, inhalable CAR-T (Chimeric Antigen Receptor-T) cell-derived exosomes loaded with PTX (PTX@CAR-Exos) achieved targeted lung accumulation in orthotopic lung-cancer mouse models, enhanced CD8^+^ T-cell infiltration, elevated IFN-γ and TNF-α levels, and improved survival with minimal toxicity [161,[163], [164], [165]]. On the translational front, scalable Good Manufacturing Practice (GMP) platforms that couple tangential-flow filtration with membrane-adsorption chromatography now routinely generate clinical-grade exosome lots with high particle yields. However, heterogeneity across batches complicates standardization. Implementing MISEV2023-aligned protocols augmented by real-time Raman spectroscopy or multi-angle flow cytometry, coupled with AI-based drift detection, ensures consistent size distribution, surface marker stoichiometry, and cargo loading. Regulatory complexity arises from donor variability and divergent global frameworks, and the cost of goods limits accessibility. Emerging strategies involve hybridization with complementary modalities, such as, exosomes decorated with enzyme-cleavable PEG for microbiome-responsive release or co-delivery of microbiome-modulating agents, pointing toward integrated, bio-responsive nanotherapeutics capable of simultaneously addressing microbial, immune, and tumor axes [161,166].

## 4 Personalized cancer nanomedicine

**Fig. 11:**
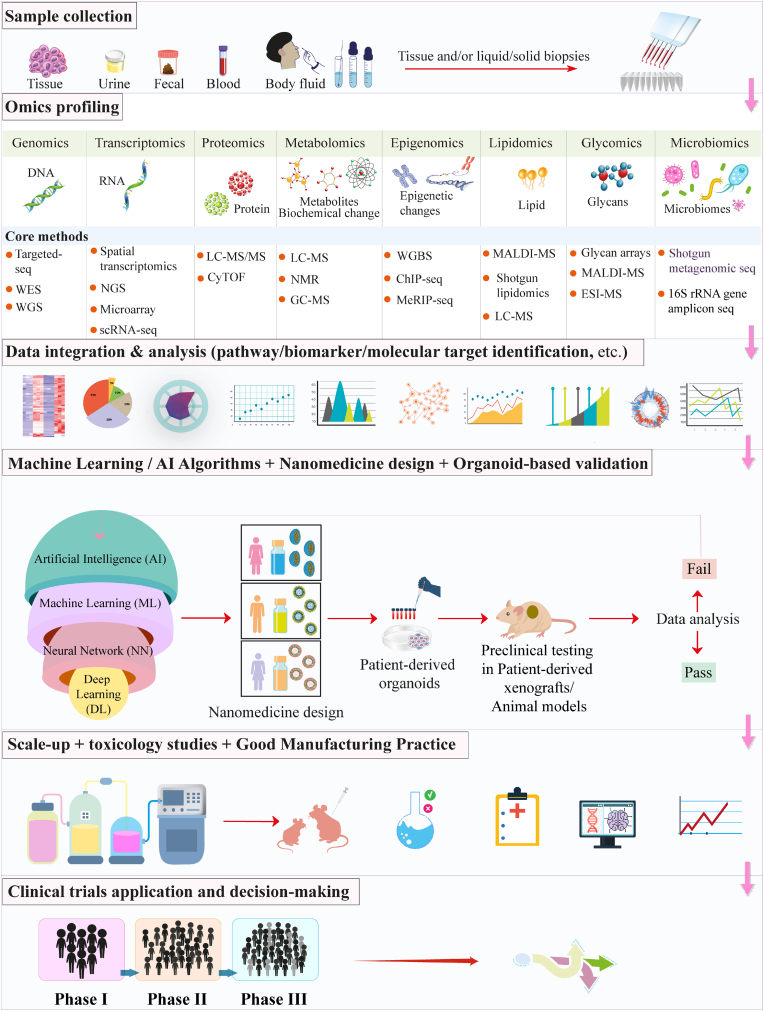
Integrated omics-driven approach for personalized cancer nanomedicine: From sample collection to clinical decision-making. (DNA – Deoxyribonucleic Acid, RNA – Ribonucleic Acid, Targeted seq – Targeted sequencing, WGBS – Whole-Genome Bisulfite Sequencing, WES – Whole-Exome Sequencing, WGS – Whole-Genome Sequencing, NGS – Next-Generation Sequencing, ChIP-seq – Chromatin Immunoprecipitation Sequencing, scRNA-seq – Single-Cell RNA Sequencing, MeRIP-seq – Methylated-RNA Immunoprecipitation Sequencing, LC-MS/MS – Liquid Chromatography Tandem Mass Spectrometry, LC-MS – Liquid Chromatography Mass Spectrometry, MALDI-MS – Matrix-Assisted Laser Desorption/Ionization Mass Spectrometry, ESI-MS – Electrospray Ionization Mass Spectrometry, GC-MS – Gas Chromatography-Mass Spectrometry, NMR – Nuclear Magnetic Resonance, CyTOF – Cytometry by Time-of-Flight, 16S rRNA seq – 16S ribosomal RNA gene sequencing, Shotgun Seq – Shotgun metagenomic sequencing.

**Fig. 12:**
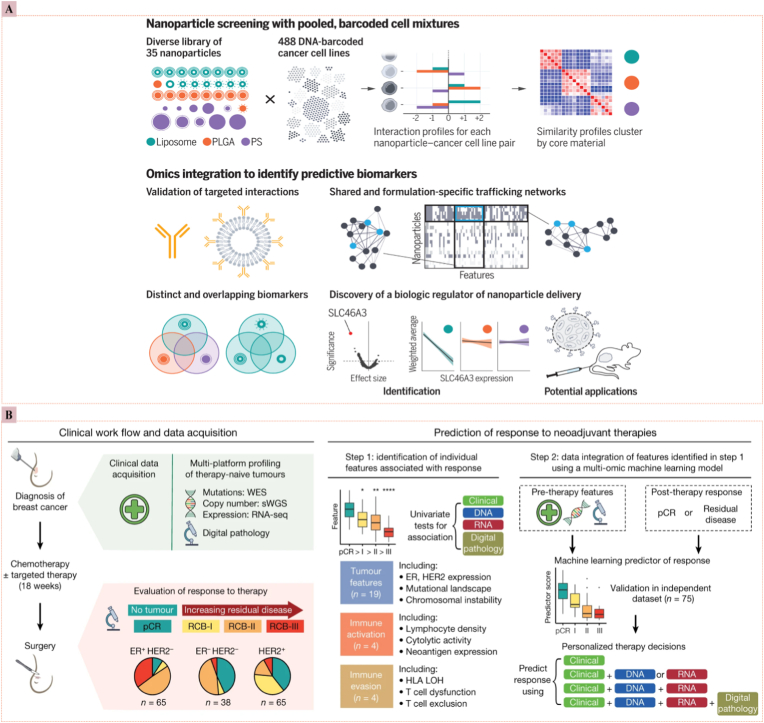
**(A)** NanoPRISM screen links of nanoparticle delivery to cancer cell omics. A curated library reveals biomarkers guiding uptake, maps trafficking networks, and uncovers a lipid-nanoparticle regulator. (PLGA, polylactide-co-glycolide; PS, polystyrene) [178]. **(B)** Pre-therapy breast tumors underwent DNA-seq, RNA-seq, and digital pathology. Post-neoadjuvant RCB response was used to train ML models on clinical, molecular, and imaging features for pCR prediction, validated in an independent cohort [181]. (sWGS, shallow whole-genome sequencing; WES, whole-exome sequencing).

**Fig. 13:**
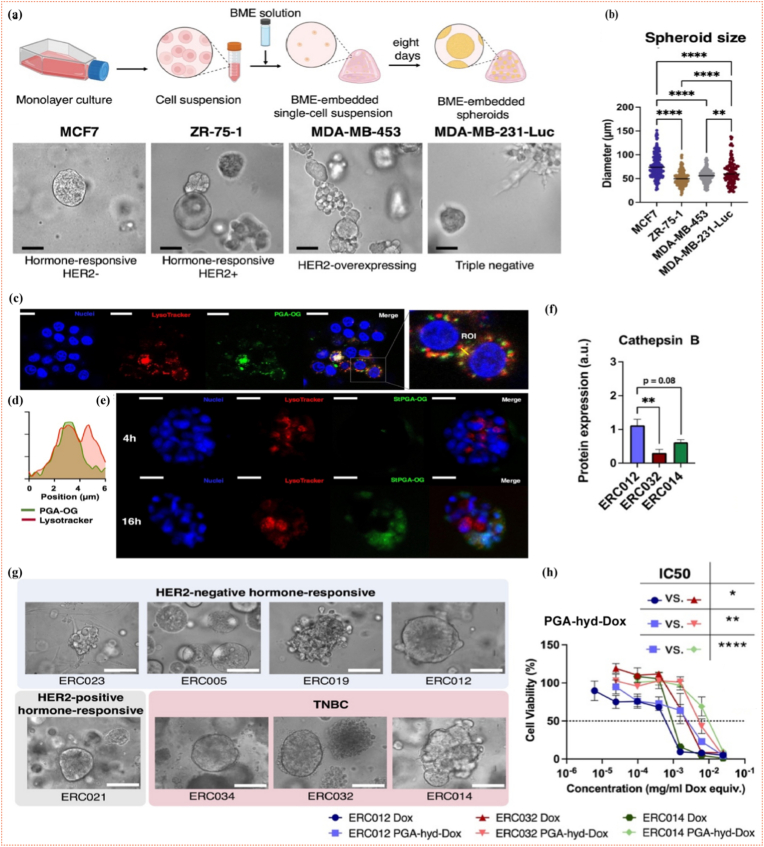
(a) Schematic workflow for fabricating BME-embedded breast cancer spheroids from single-cell suspensions and Bright-field micrographs of representative spheroids (Scale bar: 50 μm). (b) Size scatter plot of spheroids. (c) MCF7 spheroids after 4 h with linear poly-L-glutamic acid (PGA) nanocarrier conjugated to Oregon Green (green); ROI (yellow) for ImageJ line scan. (d) Fluorescence intensity of signal-peak overlap quantified by ImageJ within the ROI. (e) Time-course uptake of star-shaped PGA nanocarrier conjugated to Oregon Green (green), Lysotracker (red), nuclei labeled with Hoechst (blue); yellow = co-localization. (f) Cathepsin B/β-actin quantification (Western blot) (g) Patient-derived organoids of each subtype showing distinct morphologies (Scale bar:100 μm) (h) Cell-viability curves of ERC012, ERC014, and ERC032 organoids treated with free doxorubicin (Dox) or PGA-conjugated doxorubicin through hydrazone linker (PGA-hyd-Dox). Concentrations were chosen as Dox-equivalent doses (dox equiv.) based on drug loading [185]. (For interpretation of the references to color in this figure legend, the reader is referred to the Web version of this article.)

**Fig. 14:**
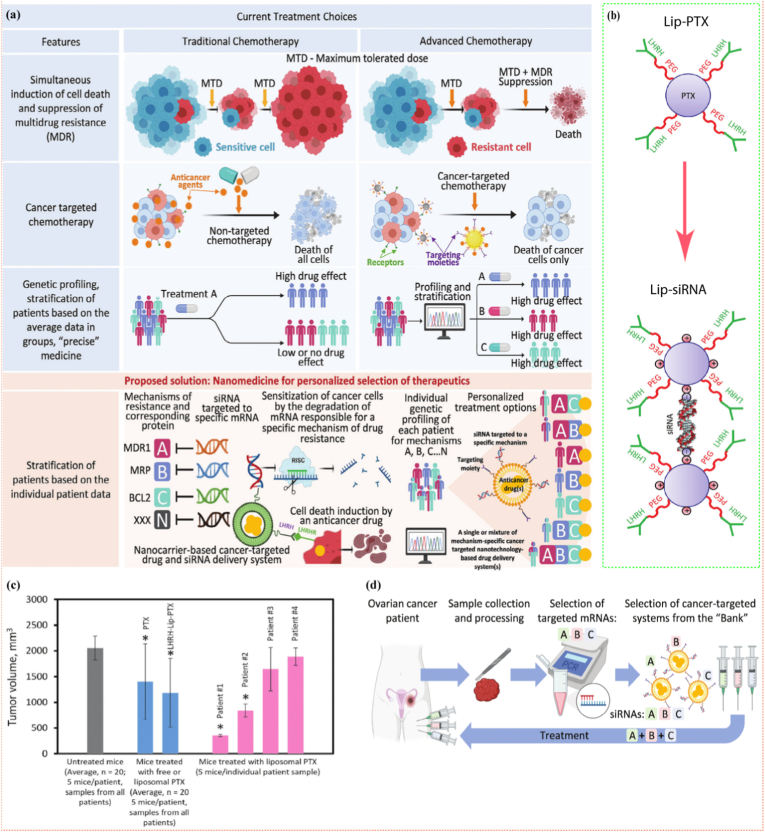
(a) From one-size-fits-all to patient-specific: the trajectory of cancer therapy. (b) Targeted nanocarriers: neutral PEGylated (PEG)–liposomes for paclitaxel (Lip-PTX) and cationic PEG–liposomes for siRNA (Lip-siRNA) (c) Tumor response overview: pooled averages vs. individual profiles of four ovarian-cancer patients; comparison of untreated, free PTX (paclitaxel), and luteinizing hormone-releasing hormone (LHRH) targeted -Lip-PTX (liposomal paclitaxel) groups (mean ± SD; p < 0.05 vs. untreated). (d) Blueprint for a personalized nanomedicine regimen in ovarian cancer [186].

Solid tumors are mosaics of genetically and phenotypically distinct sub-clones that differ inter-patient, within the same lesion (intra-tumor), and across metastatic sites (inter-lesional). This heterogeneity dictates variable nano-carrier extravasation, distribution, and pharmacodynamics, explaining why “one-size-fits-all” nanomedicines rarely achieve uniform therapeutic windows. Integrating patient-specific multi-omics datasets (genomics, epigenomics, transcriptomics, proteomics, metabolomics, lipidomics, glycomics, and microbiomics) (Table 1), with artificial intelligence (AI) guided nano-design pipelines, is now viewed as the most rational route to overcome these limitations (Fig. 11). Recent technological advances have rendered tumor heterogeneity tractable at unprecedented resolution. Integrated multi-omics workflows combining bulk and single-cell RNA sequencing, spatial transcriptomics, single-nucleus ATAC-seq, global proteomics, and multiplexed imaging enable simultaneous quantification of tumor-intrinsic drivers, immune micro-environmental signatures, and stromal barriers. These integrated data provide the foundational layer for AI/machine learning (ML) models that predict nanoparticle design, transport, uptake, and therapeutic response. Before clinical deployment, however, these models must be rigorously validated on large, independent patient cohorts to ensure robustness and generalizability. Embedding AI/ML into nanomedicine workflows converts empirical optimization into a prospective, patient-tailored design. When datasets are mapped onto pharmacokinetic–pharmacodynamic (PK–PD) models of nanoparticle behavior, they can yield quantitative rules for selecting (i) optimal carrier size, shape, and surface chemistry, (ii) tumor-restricted ligands or antibodies, and (iii) stimulus-responsive release modalities tailored to intra-tumoral pH, redox potential, or enzymatic milieu. ML algorithms, such as Random Forests (RF), Support Vector Machines (SVMs), and Gradient-Boosting Trees, are widely applied for feature selection and patient stratification in this context, classifying multi-omics and clinical signatures that link to therapeutic response. Deep-learning (DL) (specialized branch of ML) architectures, including Artificial Neural Networks (ANNs) and Convolutional Neural Networks (CNNs), integrate transcriptomic, proteomic, and radiomic datasets to predict nanoparticle biodistribution, tumor penetration, and immune modulation across heterogeneous TME. In parallel, Generative Adversarial Networks (GANs) and Variational Autoencoders (VAEs) facilitate the design of *de novo* nanocarriers and drug-like molecules, generating polymeric or lipidic formulations optimized for physicochemical stability and target selectivity [[167], [168], [169]]. Reinforcement-learning algorithms further establish an adaptive, closed-loop optimization system in which *in silico* predictions are refined through feedback from organoid-based assays, continuously converging on patient-specific design optima [170]. The predictive performance of AI-guided nanomedicine relies on high-quality, multidimensional data sets. Foundational repositories such as TCGA (The Cancer Genome Atlas), COSMIC (Catalogue Of Somatic Mutations In Cancer), DepMap (Dependency Map), and CCLE (Cancer Cell Line Encyclopedia) supply large-scale genomic, transcriptomic, and pharmacologic data, while Clinical Proteomic Tumor Analysis Consortium (CPTAC) further provides deep proteomic measurements; together, these resources are widely used to train and validate the *in-silico* component of AI-nanomedicine models [171,172]. Additional layers, including Genotype-Tissue Expression (GTEx) for tissue-specific expression, single-cell and imaging databases (e.g., the Single Cell Portal) for spatial resolution, and ClinicalTrials.gov for linking formulation parameters to therapeutic outcomes, collectively enhance the depth and context of AI training datasets [[173], [174], [175], [176], [177]]. Integration of these sources enables the construction of comprehensive AI/ML pipelines that map tumor heterogeneity onto actionable nanocarrier design rules. The practical implementation of this vision is increasingly supported by pioneering studies that leverage specific ML algorithms and robust data sources. For Instance, *Boehnke* et al. demonstrate the power of data-centric nanomedicine by coupling a massively parallel, bar-coded cell-line screen with multi-omics annotation and random forest modeling. Across 35 distinct nanoparticle formulations, the algorithm distilled genomic features into a single, core-specific predictor, the lysosomal transporter SLC46A3 (solute carrier family 46, member 3, a biomarker), whose expression inversely predicted liposomal (but not polymeric) uptake *in vitro*. CRISPR knock-out/over-expression swapped intracellular nanoparticle levels by up to 3-fold, and the same metric predicted 24 h retention of clinically approved PEGylated liposomes inside mouse tumors after intravenous dosing. The study therefore provides an example of ML translating high-dimensional tumor biology into a actionable rule: low SLC46A3 tumors receive markedly higher nanoparticle exposure, enabling prospective patient stratification for homogeneous, image-verified nanoparticle distribution (Fig. 12A) [178]. *Zhu* et al. and co-workers [179] interrogated the long-standing EPR paradigm by coupling protein nanoprobes to an AI-driven image-segmentation workflow (nano-ISML). Mapping >67000 individual vessels across 32 tumor models, they recorded a 13-fold spread in the proportion of highly leaky vessels and a 100-fold range in nanoparticle penetration between the leakiest and tightest segments. High-leakage sites favored passive extravasation, whereas low-leakage sites relied on active trans-endothelial shuttling. Guided by these data, the authors engineered genetically tuned protein nanoparticles that specifically boost transport across tight vasculature, illustrating how vessel-level permeability metrics can inform the rational design of future cancer nanomedicines. Similarly, *Ma* et al. integrated nanoparticle attributes with tumor genomic data to train an XGBoost model augmented by SHAP explainability for predicting *in vivo* nanoparticle accumulation. Across 162 literature-mined samples covering 23 cancer cell lines, the pipeline attained R^2^ scores of 0.66, 0.75, and 0.54 for delivery efficiency at 24 h, peak delivery (at any time), and 168 h, respectively. SHAP profiling revealed that tumor mass, nanoparticle diameter, and surface charge were the primary drivers of predictions, while selected somatic mutations (e.g., DNAAF1, ATP10A) contributed ∼20 % of the total feature influence. Dependence plots further revealed mutation-dependent modulation of size or zeta-potential effects, underscoring tumor genetics as a co-determinant of nanoparticles' fate. Enrichment analysis linked the implicated genes to angiogenesis and inflammatory pathways, aligning genomic features with the biological basis of the EPR effect and advocating genotype-aware design of precision nanomedicines [180]. *Sammut* et al. demonstrated that the pre-therapy tumor ecosystem's multi-omics landscape is a powerful determinant of neoadjuvant treatment response in breast cancer. By profiling pre-treatment biopsies from 168 patients, they identified that features such as genomic instability, proliferative activity, immune cell infiltration, and mechanisms of immune evasion were monotonically associated with the degree of residual disease. Integrating these clinical, digital pathology, genomic, and transcriptomic features into an ensemble ML model yielded a highly accurate predictor of pathological complete response, which was robustly validated in an independent cohort (AUC = 0.87). This work establishes a paradigm for using multi-omic data integration and ML to forecast therapeutic outcomes (Fig. 12B) [181]. Although AI-guided nanomedicine design is increasingly fueled by genomics, transcriptomics, proteomics, metabolomics, glycomics, lipidomics, and microbiomics, the field still faces computational and clinical hurdles that temper the vision of truly patient-specific nanoparticles. First, the sheer heterogeneity and dimensionality of multi-omic datasets encompassing tens of thousands of molecular features routinely exceed the modest number of nanoparticle formulations available, leaving models vulnerable to noise and over-fitting [178]. Second, no harmonized pre-processing pipeline yet exists. In a recent study, it was observed that even minor differences in single-cell RNA-seq or LC-MS/MS proteomic normalization propagated noise into ML models of tumor vascular permeability, reducing predictive R^2^ by up to 0.15 [179]. Third, the clinical logistics are daunting: generating matched whole-exome, transcriptome, proteome, metabolome, and microbiome data for a single patient remains prohibitively expensive and time-consuming, with routine turnaround times that frequently outpace the narrow therapeutic decision windows required in everyday oncology practice [22,182]. Finally, ethical and regulatory frameworks lag: merging germ-line genomic records with dynamic tumor microbiome datasets raises unresolved privacy, consent, and cross-border data-sharing issues that regulators have yet to standardize. Recognizing these constraints highlights that current AI-guided nano-designs remain largely hypothesis-generating rather than clinic-ready. It supports the pragmatic use of cost-effective, reduced-dimension biomarker panels until the aforementioned integration barriers are addressed [183,184]. Further, to translate AI-designed formulations into personalized therapies, candidate nanomedicines are first validated in patient-derived organoids (PDOs) and then extended to patient-derived xenografts (PDX) and complementary animal models to capture both cell-autonomous and systemic responses. For instance, *Boix-Montesinos* et al. [185] engineered basement-membrane-embedded 3D spheroids and a living biobank of patient-derived breast cancer organoids that faithfully recapitulate clinical inter- and intra-tumor diversity. Across these models, they measured micro-environmental traits, such as cathepsin B activity, glutathione redox state, ROS, and cytoplasmic pH. They linked them to the performance of two distinct polypeptide drug conjugates. Tumors with a high reduced/oxidized glutathione ratio showed superior responses to disulfide-bonded nanoconjugates. In contrast, elevated cathepsin B levels predicted greater sensitivity to poly-L-glutamic acid (PGA)-linked DOX. These findings demonstrate how organoid-based profiling can translate tumor-specific biology into actionable rules for selecting stimulus-responsive nanomedicines, thereby advancing precision oncology beyond generic formulations and highlighting the decisive role of PDO profiling in precision-based formulations (Fig. 13). In another research*, Garbuzenko* et al. [186] introduce a groundbreaking approach to ovarian cancer treatment by designing personalized nanomedicine. The researchers developed a complex nanocarrier system including liposomal formulations for chemotherapeutic drugs and siRNAs. These liposomes were decorated with a synthetic analog of the luteinizing hormone-releasing hormone (LHRH) peptide, which targets LHRH receptors overexpressed in ovarian cancer cells. The study validated this approach using an orthotopic PDX (mouse) model of human ovarian carcinoma, derived from cancer cells isolated from patients' tumors. This study demonstrated superior efficacy compared to conventional chemotherapy, confirming that PDO-PDX continuity can guide truly personalized nanotherapy (Fig. 14). Despite these successes, the predictive power of PDOs for nanomedicine screening must be considered in light of their inherent limitations. A primary consideration is their inability to fully recapitulate the complex TME of primary tumors, particularly the absence of functional vasculature, immune populations, and stromal components that critically influence nanoparticle transport, penetration, and overall therapeutic efficacy [187,188]. Consequently, while PDOs excel at modeling cell-autonomous drug responses, they cannot model phenomena dependent on an intact TME, such as the EPR effect or the activity of immune-modulating nanotherapies. Furthermore, the mechanical properties and interstitial pressure gradients found *in vivo* are often absent in organoid cultures, potentially leading to overestimation of nanoparticle penetration and distribution. There is also a risk of selection bias during the organoid establishment process, where the culture conditions may favor the expansion of specific subclones, thereby failing to capture the full heterogeneity of the original patient tumor [189,190]. To bridge this translational gap, future work should focus on engineering more sophisticated PDO systems co-cultured with immune and stromal cells, as well as on integrating organoid data with other preclinical models, such as PDX, to build a more holistic and predictive picture of nanomedicine performance [191,192]. Furthermore, if organoid-based testing is successful, personalized nanomedicine advances to scale-up, including Good Laboratory Practice (GLP) toxicology, GMP manufacturing, and adaptive Phase I–III clinical trials; if it fails, the data loop back to retrain the AI and redesign the formulation (Fig. 11).

**Table 1:** Summary of omics-based approaches in cancer nanomedicine [182].

Omics Technology	Analyte Focus	Key Techniques	Key nano-design parameters informed	Application in Cancer Nanomedicine	Cancer Therapy and exemplary molecular targets	Strengths	Challenges	Future Developments
**Genomics**	DNA sequence	WES, WGS, Targeted Seq	Carrier size & shape matched to EPR pore-size distribution derived from whole-exome sequencing of tumor vasculature genes (e.g., VEGFA, ANGPT2)	Personalized therapy, biomarker identification	Targeted drug delivery via mutation identification (e.g., KRAS, G12D EGFR)	High-throughput sequencing, detection of structural variations	Algorithmic complexity	Integration with AI for predictive analytics
**Epigenomics**	Modifications of DNA and histone proteins, such as methylation, acetylation, phosphorylation, etc.	ChIP-seq, WGBS, MeRIP-seq	Stimulus-responsive polymers for on-demand release of epigenetic drugs in regions of high 5-hmC or H3K27ac	Targeting epigenetic changes	Delivery of epigenetic modulators (e.g., DNMT3A-mutant hyper-methylated promoters)	Dynamic modulation, high specificity	Off-target effects, complex interactions	CRISPR-based epigenetic editing in nanoparticles
**Transcriptomics**	RNA transcripts	Microarray, NGS, scRNA-seq	Ligand selection (antibody, aptamer, peptide) based on gene expression analysis from single-cell RNA-seq	Molecular target identification, tumor heterogeneity analysis	Gene silencing nanomedicines (e.g., RNAi, CRISPR)	Comprehensive profiling, single-cell resolution	Data processing complexity	Long-read sequencing for complete transcript analysis
**Proteomics**	Proteins	LC-MS/MS, MSI, CyTOF	Protein-corona “fingerprints” from LC-MS/MS used to pre-coat nanoparticles with dysopsonins for improved circulation	Protein corona characterization	Receptor-targeted nanoparticles (e.g., HER2, EGFR)	Direct protein quantitation, high dynamic range	Sample preparation complexity, limited throughput	Proteogenomics for integrated proteome-genomic analysis
**Metabolomics**	Metabolites	NMR, GC-MS, LC-MS	Redox-sensitive linkers tuned to tumor glutathione or lactate levels measured by GC-MS.	Metabolic fingerprinting	Metabolic pathway-targeted nanoparticles (e.g., Warburg effect)	Sensitivity, precision, and reflection of cellular function	Limited metabolome coverage	Metabolite imaging for spatial resolution
**Lipidomics**	Lipids	LC-MS, MALDI-MS, Shotgun Lipidomics	Lipid raft composition guides the choice of lipidoid vs. phosphatidylcholine carriers for membrane fusion.	Lipid biomarker identification	Targeting membrane dynamics and lipid biomarkers (Cholesterol-rich lipid rafts)	Detection of lipid alterations	Lipid classification complexity	Lipid nanovesicles for targeted drug delivery
**Glycomics**	Glycans	ESI-MS, MALDI-MS, Glycan Arrays	Glycan-lectin interactions steer targeting moieties (e.g., mannose-decorated carriers for MGL^+^ macrophages)	Glycan-receptor interactions	Glycan-based biomarker discovery and targeting (Sialyl-Tn antigens)	Insights into the diversity of glycan structures contribute to the development of diagnostic tools	Sample preparation complexity	Advanced glycan microarrays for high-throughput analysis
**Microbiomics**	Microbiome	16S rRNA seq, Shotgun Seq	Intratumor microbiota profiles dictate muco-penetrating vs. muco-adhesive nano-surfaces	Cancer microbiome modulation	Microbiome-targeted therapies (e.g., microbiome mechanisms or inflammation)	Insight into microbial diversity	Variability, contamination	Microbiome-nanoparticle interactions for immunotherapy

(WES – Whole-Exome Sequencing, WGS – Whole-Genome Sequencing, Seq – Sequencing, KRAS G12D – KRAS Glycine-to-Aspartate mutation at codon 12, EGFR – Epidermal Growth Factor Receptor, VEGFA – Vascular Endothelial Growth Factor A, ANGPT2 – Angiopoietin-2,ChIP-seq – Chromatin Immunoprecipitation Sequencing, WGBS – Whole-Genome Bisulfite Sequencing, MeRIP-seq – Methylated RNA Immunoprecipitation Sequencing, 5-hmC – 5-hydroxymethylcytosine, H3K27ac – Histone 3 Lysine 27 acetylation, DNMT3A – DNA Methyl-Transferase 3A, NGS – Next-Generation Sequencing, scRNA-seq – single-cell RNA sequencing, RNAi – RNA interference, LC-MS/MS – Liquid Chromatography tandem Mass Spectrometry, MSI – Mass-Spectrometry Imaging, CyTOF – Cytometry by Time-Of-Flight (mass cytometry), HER2 – Human Epidermal growth factor Receptor 2, NMR – Nuclear Magnetic Resonance, GC-MS – Gas Chromatography–Mass Spectrometry, LC-MS – Liquid Chromatography–Mass Spectrometry, MALDI-MS – Matrix-Assisted Laser Desorption/Ionization Mass Spectrometry, ESI-MS – Electrospray Ionization Mass Spectrometry, Shotgun Lipidomics – Direct infusion lipid analysis by MS, Glycan Arrays – Microarrays printed with diverse glycans,MGL – Macrophage Galactose-type Lectin, Sialyl-Tn – Sialylated Tn antigen (tumor-associated glycan), 16S rRNA seq – 16S ribosomal RNA gene sequencing, Shotgun Seq – Shotgun metagenomic sequencing, EPR – Enhanced Permeability and Retention (effect).

## 5 Competitive landscape of cancer nanomedicine

Nanomedicine holds immense promise, while its translational potential and unique value proposition can be better understood when contextualized alongside other pillars of precision oncology, such as antibody-drug conjugates (ADCs) and cell-based therapies, such as CAR-T cells.

### 5.1 Antibody-drug conjugates (ADCs)

ADCs represent a mature and clinically validated class of targeted therapeutics. Their "magic bullet" design, a monoclonal antibody linked to a potent cytotoxic payload, shares nanomedicine's goal of improving the therapeutic index. The primary advantages of ADCs lie in their well-defined chemistry, scalable manufacturing, and remarkable clinical success in targeting molecules such as HER2 (e.g., Trastuzumab deruxtecan) and TROP2 (e.g., Sacituzumab govitecan). Their mechanism is precise, relying on antigen binding and internalization to release the payload intracellularly, often with a potent "bystander effect" that can kill adjacent antigen-negative cells in a heterogeneous tumor [193,194]. However, ADCs face limitations that nanomedicine platforms, exceptionally engineered exosomes, aim to address. ADC toxicity is often linked to "on-target, off-tumor" effects due to low-level antigen expression on healthy tissues and the release of the highly potent payload into the bloodstream from linker instability. Furthermore, ADC efficacy is limited by the availability of target antigens and the potential for antigen escape. In contrast, nanocarriers such as exosomes offer a higher payload capacity, enabling the co-delivery of multiple therapeutic agents (e.g., drugs and siRNA) to overcome resistance pathways. Their surface can be engineered with various targeting ligands to improve specificity and penetration, and their endogenous composition may reduce immunogenicity and enhance biocompatibility compared to some ADC constructs [156,162,195,196].

### 5.2 Cell-based therapies

CAR-T cell therapy represents the pinnacle of personalized medicine, engineering a patient's own T cells to recognize and target cancer cells for eradication. Its successes, particularly in hematological malignancies like B-cell acute lymphoblastic leukemia and diffuse large B-cell lymphoma, are unprecedented, demonstrating remarkable complete response rates [197]. The key strengths of CAR-T cells are their capacity for *in vivo* expansion and long-term persistence, which can potentially lead to durable remissions and a single-dose curative effect a feature that no nanomedicine or ADC can currently claim [198]. They are "living drugs" that can adapt and respond dynamically within the TME. The challenges for CAR-T cells, however, are profound, creating a clear niche for nanomedicine. CAR-T therapy is associated with severe toxicities, such as Cytokine Release Syndrome (CRS) and Immune Effector Cell-Associated Neurotoxicity Syndrome (ICANS), which require sophisticated clinical management [199]. It remains ineffective mainly against solid tumors due to difficulties in trafficking, infiltrating, and surviving in the immunosuppressive TME, as well as antigen heterogeneity [200]. Furthermore, the process is complex, personalized, time-consuming (often taking weeks to manufacture), and extraordinarily expensive, limiting its broad accessibility [201]. This is where nanomedicine offers compelling alternatives, such as the development of 'off-the-shelf' alternatives that bypass the need for personalized cell engineering altogether. Nano-immunotherapies, such as engineered exosomes displaying T-cell engagers or checkpoint inhibitors, can be designed as pre-manufactured reagents to redirect a patient's existing immune cells *in situ*. This approach promises a superior safety profile, enhanced penetration into solid tumors, and significantly lower cost and complexity compared to autologous cell therapy [158]. Alternatively, nanomedicine can enable CAR-T therapy by overcoming its primary barrier of the immunosuppressive TME. Iron oxide nanoparticles (IONPs), for instance, can reprogram this niche by polarizing macrophages to a pro-inflammatory M1 state and disrupting metabolic barriers. This 'priming' converts 'cold' tumors into 'inflamed' environments, making them receptive to subsequent CAR-T cell infiltration and activity for a powerful combinatorial strategy [202]. Moving forward, hybrid approaches such as nanoparticle-assisted CAR-T delivery or antibody–nanoparticle conjugates represent promising strategies that integrate the precision of biologics with the versatility of nanoscale engineering, advancing the next generation of personalized cancer therapeutics [203,204].

## 6 Current status and challenges in the translation of cancer-nanomedicine

### 6.1 Current status of cancer-nanomedicine

Nanomedicine formulations have had a significant impact on the global healthcare system and pharmaceutical market. Currently, approximately 100 nanomedicine-based formulations have been approved by regulatory agencies such as the FDA and the European Medicines Agency (EMA) [205]. The number of these formulations is increasing annually, with various cancer nanomedicine iterations of previously approved drugs undergoing clinical trials to evaluate their efficacy compared to traditional dosage forms [206]. This growth is driven by increased research and development (R&D) and market demand for innovative drug delivery solutions. As summarized in Table 2, the worldwide portfolio of approved cancer nanomedicines sanctioned by the FDA, EMA, Russian Federation Ministry of Public Health (RFMPH), Korean Ministry of Food and Drug Safety (MFDS), and National Medical Products Administration (NMPA)(China) are dominated by five kinds of the nanomedicine: liposomal and lipid nanoparticles, polymeric micelles/conjugates, nanocrystal suspensions, inorganic nanoparticles (e.g., iron oxide, hafnium oxide), and albumin or recombinant protein-based nanocarriers. The clinical pipeline continues to expand robustly. As of 2021, alongside the ∼100 commercially available nanomedicines, a further 563 candidates were advancing through clinical trials or late-stage development. Over half of these trials (53 %) are Phase I/II studies, and the vast majority target oncology indications [21,205]. Recent cancer nanomedicines (as catalogued in ClinicalTrials.gov) employing passive/active targeting, or stimuli-responsive strategies, and currently undergoing various stages of clinical trials, are summarized in Table 3, Table 4, respectively. Despite this considerable investment and pipeline, the clinical translation of cancer nanomedicine has been marked by several high-profile failures that provide critical lessons for the field. A prime example is the case of MM-302, a HER2-targeted liposomal DOX. Despite a strong preclinical rationale, a Phase II trial (NCT02213744) in HER2-positive metastatic breast cancer failed to demonstrate a significant improvement in progression-free survival over standard-of-care regimens. This failure has been attributed to several factors, including insufficient proof of superior tumor targeting *in vivo* and the formidable biological barriers. Similarly, BIND-014, a prostate-specific membrane antigen (PSMA)-targeted docetaxel nanoparticle, showed promising early-phase results but failed in subsequent larger trials for prostate and lung cancers. Post-hoc analyses suggested that patient stratification based on PSMA expression was inadequate, and the anticipated enhancement of the therapeutic index was not fully realized in an unselected patient population [207,208]. Etirinotecan Pegol (NKTR-102) failed to demonstrate efficacy in a clinical trial for patients with active brain metastases. The failure was attributed to an inability to cross the intact BBB, resulting in no significant drug accumulation in the EPR-negative brain tumors [209]. These examples underscore that nanomedicine failures often stem from biological heterogeneity rather than flawed nanochemistry, therefore necessitating the development of more predictive preclinical models and robust biomarkers for effective patient stratification in clinical trials.

**Table 2:** Representation of selective cancer nanomedicines sanctioned by regulatory bodies such as the FDA, EMA, RFMPH, MFDS, and NMPA.

Clinical Indication	Trade Name	Manufacturer	Year of Approval	Regulatory Agency	Key nanocarrier	Active Ingredient	Administration Route	Ref.
Metastatic pancreatic adenocarcinoma	Onivyde®	Merrimack Pharmaceuticals, Cambridge, UK	2015	FDA	Liposome	Irinotecan	Intravenous	[210,211]
Breast cancer	Myocet®	Teva Pharmaceutical Industries Ltd, Petah Tikva, Israel	2000	EMA	Liposome	Doxorubicin	Intravenous	[212]
Breast, advanced non-small cell lung, pancreatic cancer	ABRAXANE®	Abraxis Bioscience, Bristol-Myers Squibb®, NJ, USA	2005	FDA	Paclitaxel albumin-bound particles	Paclitaxel	Intravenous	[213]
Ovarian cancer, Kaposi's sarcoma, multiple myeloma	Doxil®	Johnson and Johnson, NJ, USA	1995-FDA, 1996-EMA	FDA, EMA	Liposome	Doxorubicin	Intravenous	[214,215]
Ovarian cancer, fallopian tube cancer, peritoneal cancer	Paclical®	Oasmia Pharmaceuticals AB, Uppsala, Sweden	2015	RFMPH	Micelle	Paclitaxel	Intravenous	[216]
Ovarian cancer, peritoneal cancer, fallopian tube cancer	Apealea®	Oasmia Pharmaceuticals AB, Uppsala, Sweden	2018	FDA	Micelle	Paclitaxel	Intravenous	[217]
AIDS-associated Kaposi's sarcoma	DaunoXome®	Galen Pharmaceuticals, USA	1996	FDA, EMA	Liposome	Daunorubicin citrate	Intravenous	[218,219]
Neoplastic or Lymphomatous Meningitis	Depocyt®	Skye Pharm Inc. CA, USA	1999-FDA, 2001-EMA	FDA, EMA	Liposome	Cytarabine	Intrathecal	[220,221]
Breast cancer, ovarian cancer, Kaposi's sarcoma	Caelyx®	Janssen Pharmaceutica NV	1996	EMA	PEGylated liposome	Doxorubicin	Intravenous	[222,223]
Locally advanced soft tissue sarcoma	Hensify®	Nanobiotix, Paris, France	2019	EMA	Hafnium oxide nanoparticle	Hafnium oxide	Local Injection	[205]
Bone cancer	Mepact®	Takeda Pharmaceutical Company Limited, Osaka, Japan	2009	EMA	Liposome	Mifamurtide	Intravenous	[224]
Philadelphia chromosome-negative acute lymphoblastic leukemia	Marqibo®	Talon Therapeutics, CA, USA	2012	FDA Withdrawal in 2022	Liposome	Vincristine	Intravenous	[225,226]
AIDS-related Kaposi's sarcoma, ovarian cancer, multiple myeloma	Lipodox®	Sun Pharmaceutical Industries Ltd.	2013	FDA	Liposome	Doxorubicin	Intramuscular/Intravenous	[227]
Acute lymphoblastic leukemia	Oncaspar®	Enzon Pharmaceuticals Inc	FDA-2006, EMA 2016	FDA, EMA	Polymeric/PEGylated enzyme	Pegaspargase	Intravenous	[228]
Gastric cancer	Liporaxel® /DHP107	Daewoong Pharmaceutical Co., Ltd, South Korea	2016	MFDS	Paclitaxel lipid nanoparticles	Paclitaxel	Oral	[229]
Breast cancer, non-small-cell lung, ovarian, gastric cancer	Genexol-PM®	Samyang Pharmaceuticals, Seoul, South Korea	2007	MFDS	Micelle	Paclitaxel	Intravenous	[230]
Lung squamous cell carcinoma, breast cancer, ovarian cancer	Lipusu®	Luye Pharma, Nanjing, China	2003-NMPA, 2016-FDA	NMPA FDA	Liposome	Paclitaxel	Intravenous	[213,231,232]
Cutaneous T cell lymphoma, leukemia,	Ontak®	Eisai Co., Ltd, Tokyo, Japan	1999	FDA	Recombinant fusion protein-based nanoformulation	Denileukin diftitox	Intravenous	[233]
High-risk acute myeloid leukemia	Vyxeos®	Jazz Pharmaceutics, CA, USA	FDA-2017, EMA-2018	FDA, EMA	Liposome	Daunorubicin and cytarabine	Intravenous	[234]
Brain tumor	NanoTherm®	Magforce Ag, Berlin, Germany	2011	EMA	Iron oxide nanoparticles	Iron oxide as a functional agent	Intratumoral	[98]
Pancreatic cancer	ONIVYDE®	Merrimack Pharmaceuticals, Massachusetts, USA	2015	FDA	Liposomal irinotecan	Irinotecan	Intravenous	[235]

**Table 3:** Representative nanomedicines (passive and/or active targeting) undergoing various stages of clinical trials for oncological applications (ClinicalTrials.gov) [21,205,216,218,[236], [237], [238]].

Clinical Indication	Trade Name/Code/Other Name	NCT Number	Clinical phase, Status	Key nanocarrier	Active Ingredients/drug product
Breast carcinoma	Cytoxon	NCT00629499	Phase II, Completed	Albumin-based Nanoparticles	Cyclophosphamide/Paclitaxel
Breast cancer	NK-105	NCT01644890	Phase III, Completed	Polymeric micellar nanoparticle	Paclitaxel
Metastatic triple-negative breast cancer	ML39079_ALICE	NCT03164993	Phase II, Completed	PEGylated liposomes	Atezolizumab, Doxorubicin, Cyclophosphamide
Advanced solid tumors, Metastatic colorectal cancer	LE-SN38-101/CALGB-80402	NCT00046540/NCT00311610	Phase I, Completed/Phase II, Completed	Liposome	Active metabolite of irinotecan (SN-38)
Metastatic Breast Cancer	NKTR-102	NCT02915744	Phase II, Completed	PEGylated liposomes	Irinotecan
Metastatic pancreatic cancer with KrasG12D mutation	iExosomes	NCT03608631	Phase I, Recruiting	Exosomes	Mesenchymal stromal-derived cells with KRAS G12D small interfering RNA (siRNA)
Cutaneous squamous cell carcinoma skin cancer	STP705	NCT04844983	Phase II, Completed	Polymeric nanoparticles	siRNA
Stage IV lung cancer, Advanced lung cancer	ONC-002/ONC-003	NCT01455389/NCT04486833	Phase I & II, Terminated/Phase I & II, Recruiting	Lipid-based nanoparticles	TUSC2 tumor suppressor gene/DNA plasmid with the TUSC2 tumor suppressor gene
Melanoma	Allovectin-7®	NCT00395070	Phase III, Completed	Lipid-based nanoparticles	VCL-1005 DNA plasmid
Pediatric recurrent or refractory Solid tumors/Paediatric recurrent or progressive CNS tumors/Metastatic pancreatic cancer	SGT-53/SGT53-00-1/SGT53-02-1	NCT02354547/NCT03554707/NCT02340117	Phase I, Suspended/Early Phase I, Unknown status/Phase II, Active, not recruiting	Liposomes	DNA encoding the p53 tumor suppressor gene
Non-small-cell lung cancer	EMR 63325-001	NCT00409188	Phase II, Completed	Liposomes	Mucinous glycoprotein 1 (MUC1) antigen
Melanoma	MAGE-A3+AS15	NCT00796445	Phase III, Terminated	Liposomes	Human melanoma-associated antigen A3 (MAGE-A3) protein + immunostimulant adjuvant AS15
Non-small-cell lung cancer	MAGE-A3+AS15	NCT00480025	Phase III, Terminated	Liposomes	MAGE-A3 + AS15
Mesothelioma, non-small-cell lung cancer	TargomiRs	NCT02369198	Phase I, Completed	Nanoparticles with an anti-epidermal growth factor receptor bispecific antibody	Double-stranded miR-16 microRNA mimic
Breast cancer	MM-302	NCT02213744	Phase I & III, Terminated	PEGylated liposomes	Doxorubicin
Malignant melanoma/Advanced Lung Cancer	Taxoprexin	NCT00087776/NCT00243867	Phase III, Terminated/Phase III, Completed	Polymer-drug conjugate	Paclitaxel covalently linked to docosahexaenoic acid (DHA)/carboplatin
Advanced melanoma	Lipo-MERIT	NCT02410733	Phase I, Completed	Liposomes	Four naked antigen-encoding RNAs
Epithelial ovarian, fallopian tube or primary peritoneal cancer/Advanced solid tumors	CRLX101	NCT02389985/NCT01652079/NCT02648711	Phase I & II, Terminated/Phase II, Completed/Phase I, Terminated	Polymeric nanoparticles	Cyclodextrin camptothecin/Paclitaxel/Bevacizumab/modified FOLFOX regimen
Triple-negative breast cancer	BN_0002-01	NCT02316457	Phase I, Completed	Lipid nanoparticle	RNAs encoding patient-specific antigens
Glioblastoma or gliosarcoma	NU 16C01/NCI-2016-02007	NCT03020017	Early Phase1, Completed	Gold nanoparticles	NU-0129 (siBcl2L12-SNAs)
Pancreatic cancer	NC-6004-005	NCT02043288	Phase III, Completed	Polymeric micellar nanoparticles	Cisplatin
Ovarian, peritoneal, or fallopian tube cancer/Non-small-cell lung cancer	GOG-0212/CDR0000269910	NCT00108745/NCT00054210	Phase III, Unknown status/Phase III, Terminated	Polymeric nanoparticles	Paclitaxel
Advanced Solid Tumor	HZDH20-002	NCT04778839	Phase I/Recruiting	Micelles	Paclitaxel
Hepatic Metastases, Triple-negative breast, Pancreatic adenocarcinoma	EndoTAG® −1, EndoTAG-1+GEM	NCT00542048/NCT03002103/NCT03126435	Phase II, Completed/Phase III, Suspended/Phase III, Completed	Liposomes	Paclitaxel, Gemcitabine Hydrochloride

**Table 4:** Representative nanomedicines (stimuli responsive) undergoing various stages of clinical trials for oncological applications (ClinicalTrials.gov) [119,239,240].

Clinical Indication	Trade Name/Code/Other Name	Key nanocarrier	Stimulus or Trigger	Cargo/Therapeutic Agent	Platform/Mechanism	NCT Number	Clinical Phase, Status	Key Outcomes/Notes
Pancreatic tumors/Hepatocellular carcinoma/liver cancer	PanDox, ThermoDox®	Liposomal	Temperature/mild hyperthermia/radiofrequency	Doxorubicin	Thermosensitive liposomal doxorubicin activated by heat (∼42 °C), with radiofrequency ablation (RFA)	NCT04852367/NCT00441376/NCT02112656/NCT00617981	Phase I, Withdrawn/Phase I, Completed/Phase III, Completed/Phase III, Completed	Proof-of-concept for local heat-triggered drug release, but mixed results due to heating variability/Overall, no significant survival compared to RFA alone
Soft-tissue sarcoma of the extremity/Head & neck squamous cell cancer/Lung cancer, Soft tissue sarcoma of the extremity and trunk wall/Pancreatic cancer	NBTXR3 (Hensify®)	Inorganic nanoparticles	Ionizing radiation	None (radio-enhancer)	Hafnium-oxide nanoparticles injected intratumorally to amplify energy deposition from radiotherapy	NCT01433068/NCT04862455/NCT04505267/NCT02379845/NCT04484909	Phase I, Completed/Phase II, Active, not recruiting/Phase I, Recruiting/Phase II & III, Completed/Phase 1, Recruiting	Improved local tumor control, manageable safety/First-in-class radioenhancer
Glioblastoma/Prostate cancer	NanoTherm® (MagForce)	Magnetic nanoparticles	Alternating magnetic field	None (heat energy)	Superparamagnetic iron-oxide nanoparticles (SPION) generate localized hyperthermia	NCT06271421/NCT05010759	Interventional studies, Recruiting/Terminated	Feasibility shown in recurrent glioblastoma/Median survival benefit reported
Head and neck tumors/Lung tumor	AuroLase^TM^/AuroShells	Core–shell nanoparticle	Near-infrared (NIR) photothermal	None (photothermal conversion)	Gold–silica nanoshells converting NIR light to heat for ablation	NCT00848042/NCT01679470	Interventional study, Completed/Pilot feasibility study, Terminated	Effective localized ablation/Effective energy conversion, but limited depth
Glioblastoma	NVX108-GBM1B	Nanoemulsion	Radiation	Dodecafluoropentane (Oxygen carrier)	Perfluorocarbon nanoemulsion enhances radiation sensitization and tumor oxygenation	NCT02189109	Phase I, Completed	Enhanced oxygenation and radiosensitization/Safe in early trials
Primary and metastatic liver cancers	Radiotherapy With Iron Oxide Nanoparticles	Magnetic nanoparticles	Radiation	Ferumoxytol	Ferumoxytol (SPION) in combination with MRI-guided radiotherapy	NCT04682847	Phase I, Observational study (Active, not recruiting)	Showed promise by enhancing tumor visualization, radiomodulation, and induction of ferroptosis/Feasibility and safety of approach
Superficial basal cell carcinomas	Ameluz, Biofrontera (BF-200 ALA)	Nanoemulsion	Light Photodynamic therapy (PDT)	Aminolevulinic (photosensitizer)	Aminolevulinic acid nanoemulsion generates reactive oxygen species (ROS) upon light activation	NCT02367547	Phase I & II, Active, not recruiting	Showed an effective photosensitizer for PDT and a higher response rate/Better cosmetic outcome
Multiple brain metastases/Brain Metastases/Glioblastoma/Lung and pancreatic cancers	NANO-RAD, NANO-GBM, Nano-SMART	Inorganic hybrid nanoparticles	Radiation	Polysiloxane (silica) network core, grafted with gadolinium chelates	AGuIX Gadolinium-based nanoparticle enhances the effectiveness of radiation therapy	NCT02820454/NCT04899908/NCT04881032/NCT04789486	Phase I, Completed/Phase II, Recruiting/Phase I & II, Active, not recruiting/Phase I & II, Recruiting	Versatile and practical theranostic nanoparticle that enhances radiotherapy, tumor visualization/Significant potential in precision cancer nanomedicine
Advanced or refractory tumors	LiPlaCis	Liposomes	Enzyme	Cisplatin	Phospholipase A2-sensitive liposomes broken down by enzyme secretory phospholipase A2 (sPLA2), overexpressed in many solid tumors, and release targeted cisplatin	NCT01861496	Phase I & II, Completed	Smart and targeted therapy, but did not succeed in late-stage clinical trials/Proof-of-concept in the field of targeted nanomedicine
Non-small cell lung, pancreatic, or colorectal cancer	NBF-006-001	Lipid nanoparticles	Enzyme	siRNA	Lipid nanoparticles deliver siRNA silencing Glutathione S-Transferase Pi (GSTP) enzyme overexpressed in cancer cells	NCT03819387	Phase I, Completed	Showed early efficacy and confirmed on-target activity against GSTP in tumors/Proof-of-concept for targeted therapy
Rectal cancer	LCCC 1315	Polymeric nanoparticles	Radiation	Cyclodextrin camptothecin	Polymeric nanoparticles release camptothecin to inhibit topoisomerase I and HIF-1α, enhancing radiosensitivity	NCT02010567	Phase 1 & II, Terminated	The regimen was not well-tolerated/Excessive toxicity led to termination

### 6.2 Challenges in the translation of cancer-nanomedicine

**Fig. 15:**
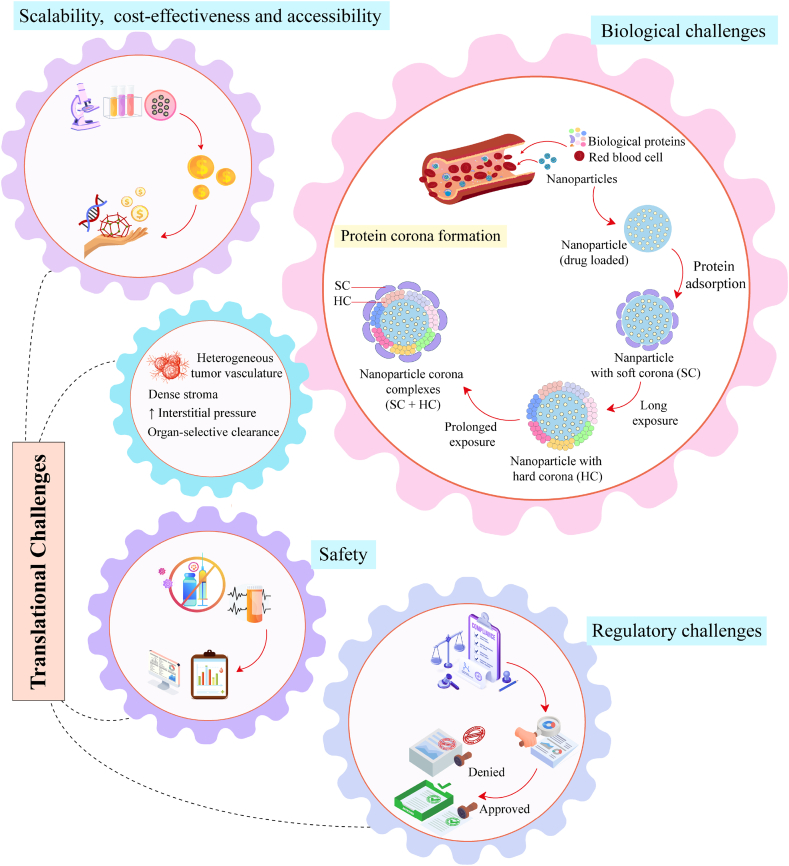
Translational challenges in cancer nanomedicine: manufacturing and commercial hurdles (scalability, cost-effectiveness, accessibility), biological and safety barriers, and regulatory challenges.

The number of nanotherapeutics in drug development rapidly increases, but clinical success remains modest. This indicates persistent issues that require attention. Although each cancer nanotherapeutic presents distinct concerns, key bottlenecks include scalability, cost, accessibility, biological challenges such as biodistribution, safety, and regulatory hurdles (Fig. 15).

#### 6.2.1 Scalability, cost-effectiveness, and accessibility

Bench-scale protocols optimized for milligrams often fail at the industrial scale, as minor changes in shear, temperature, or solvent ratios can significantly alter particle size, drug loading, and release kinetics. The capital required for GMP-grade reactors, continuous-flow microfluidics, aseptic filling, and real-time quality control escalates rapidly, forcing developers to favor incremental reformulations of already-approved drugs rather than first-in-class constructs. Rigorous cost-benefit modeling and scalable process design must begin at the earliest stage of discovery to prevent late-stage financial attrition [52]. Furthermore, the development and implementation of personalized nanomedicine involve significant costs, including research and development, manufacturing, and clinical trials. While the scientific trajectory of personalized nanomedicine is promising, its clinical translation must overcome significant economic and accessibility barriers to avoid exacerbating global health disparities. The vision of tailoring nanocarriers to individual patient omics profiles is inherently resource-intensive, requiring expensive multi-omics analyses, complex GMP processes for small-batch production, and sophisticated clinical infrastructure for administration and monitoring. These factors pose a profound challenge for low- and middle-income countries (LMICs), where healthcare systems are often strained, and resources are limited. To realize the global promise of these therapies, future efforts must parallel scientific innovation with cost-reduction strategies. This includes developing low-cost, point-of-care diagnostic platforms for patient stratification, investing in decentralized and modular manufacturing technologies to reduce production costs, and establishing harmonized and streamlined regulatory pathways to accelerate approval. Furthermore, international public-private partnerships and global health funding initiatives will be crucial to ensure that the benefits of precision oncology are equitable and accessible worldwide, moving beyond a 'one-size-fits-all' model to a 'right-size-for-all' paradigm [[241], [242], [243], [244]].

#### 6.2.2 Biological challenges

Effective nanomedicine design dictates how carriers navigate complex biological barriers and distribute within the body. Heterogeneous tumor vasculature, dense stroma, elevated interstitial pressure, and organ-specific clearance pathways collectively determine whether a formulation reaches its target or is prematurely sequestered [237,245]. Yet biodistribution is further modulated by species-specific differences in vascular physiology and protein corona. Protein corona formation is a dynamic process where proteins from biological fluids adsorb onto the surface of nanoparticles, forming Soft and hard corona (SC and HC) (Fig. 15). This process varies significantly across different nanomaterials due to differences in size, shape, surface chemistry, and the nature of the biological environment. For instance, metal nanoparticles like gold and silver form dense, stable coronas through strong metal-protein interactions [246], while metal oxides like TiO_2_ or Fe_3_O_4_ exhibit charge and pH-dependent adsorption via surface hydroxyl groups [247]. Polymeric nanoparticles exhibit hydrophobic-driven binding; however, surface modifications, such as PEGylation, can reduce protein adsorption, resulting in a softer, more dynamic corona [248]. Carbon-based materials (e.g., graphene, carbon nanotubes) interact primarily through π–π and hydrophobic forces, often leading to protein conformational changes [249]. Quantum dots form unstable coronas unless coated [250], whereas lipid nanoparticles develop soft, reversible coronas enriched in apolipoproteins that influence biodistribution [251]. The protein corona determines the fate of nanocarriers by altering their circulation, targeting, and immune response, which can reduce efficacy and introduce toxicity. To mitigate the negative impacts of protein corona formation, several design strategies can be employed. Passive Shielding involves functionalizing nanoparticles with specific ligands or polymers, such as PEG or zwitterionic polymers, to reduce non-specific protein adsorption and create a more dynamic corona [252]. Active surface functionalization can be achieved by pre-incubating nanoparticles with specific proteins to form a pre-formed corona that enhances targeting and reduces off-target effects. Additionally, designing high-density, oriented displays of targeting ligands (e.g., peptides, antibodies) on long spacer arms can improve their accessibility and binding efficiency despite corona formation [253,254]. Optimizing core properties by tuning the size and shape of nanoparticles can influence the corona's stability and composition, with smaller, highly curved particles fostering a more dynamic corona compared to larger, flatter surfaces. In some cases, external forces such as magnetic fields can be used to physically direct nanoparticles to the target site, thereby overriding the biodistribution dictated by the corona. An integrated design approach that combines these strategies is essential for developing effective nanomedicines [255,256]. Furthermore, comprehensive mapping of nanoparticle interactions via quantitative imaging and mass-balance techniques is thus indispensable for bridging pre-clinical results to predictable clinical performance [257].

#### 6.2.3 Safety

After i.v. administration, nanocarriers rapidly acquire a protein corona, undergo non-specific uptake by the mononuclear phagocyte system, and must traverse irregular tumor vasculature, dense extracellular matrix, and high interstitial pressure. These hurdles shorten the half-life and lower the intra-tumoral exposure [237,245,258]. Standard conventional toxicology assessments, initially designed for small molecules, often overlook nanospecific attributes (size, shape, surface chemistry, and aggregation state) that critically modulate biodistribution and immunogenicity. Therefore, formulation changes (coating, ligand density, synthetic route) can unpredictably alter the safety profile and mandate new GLP studies [23,[259], [260], [261]]. Moreover, a particularly nuanced safety challenge emerges from the very promise of multi-modal nanomedicines, especially those combining chemotherapy with immunostimulatory agents (e.g., checkpoint inhibitors) and physical therapies (e.g., photothermal ablation). While designed for synergistic anti-cancer efficacy, these combinations can also precipitate synergistic toxicity and severe immune-related adverse events (irAEs) [262]. For instance, ICD induced by chemo or photothermal therapy can lead to a massive release of pro-inflammatory cytokines (a "cytokine storm"), while concurrently administered immunoadjuvants may amplify this response, potentially causing systemic inflammatory syndrome [263]. Furthermore, the abscopal effect, where local treatment triggers a systemic immune response, can inadvertently lead to autoimmune attacks on healthy tissues [264]. Managing these complex toxicity profiles requires moving beyond conventional safety paradigms. Future development must focus on predictive biomarkers for patient stratification, temporal control over the release of combination agents to de-couple efficacy from toxicity, and the implementation of novel toxicity endpoints in clinical trials that specifically monitor for irAEs and synergistic organ damage [265,266].

#### 6.2.4 Regulatory challenges

A significant roadblock remains a lack of harmonized standards for manufacturing, quality control, safety, and efficacy testing. No international regulatory standards are specially designed for the clinical translation of nanomedicine [241,242,267]. Regulatory bodies, such as the FDA and the EMA, have issued initial guidance documents to provide direction on the use of nanotechnology and nanomaterials. However, these documents are not legally binding and merely represent the FDA's or EMA's current thinking. In April 2022, the FDA released the final guidelines for pharmaceutical goods, including biological products that use nanomaterials. Notwithstanding these attempts, some concerns remain unsettled, urging industry enterprises to seek more clarification and data. A crucial regulatory challenge is the inconsistency in how various geographical areas address nanotechnology applications. This variation is noticeable in cases where nanomedicine that has received approval in a specific country is denied approval in others. To promote the effective advancement of nanomedicine, it is essential to standardize regulatory standards across various regions [261,267]. Establishing unified regulations presents a significant challenge; however, it is crucial for the advancement of nanomedicine.

## 7 Future perspective

The future of cancer therapy lies in precisely engineered, patient-centric nanomedicine based platforms that transcend the limitations of empirical, population-based dosing. Next-generation systems should synergistically integrate (i) multi-modal targeting, combining passive EPR exploitation with active ligand–receptor engagement, (ii) stimulus-responsive release calibrated to the unique biochemical signatures of an individual TME, and (iii) artificial intelligence (AI)-driven design loops that continuously refine nanoparticle architecture against real-time multi-omics data. Such convergent strategies promise to maximize intra-tumoral drug exposure while minimizing systemic toxicity, thereby redefining therapeutic indices in clinical oncology.

### 7.1 Beyond the "one size fits all" approach

Inter-patient heterogeneity in vascular architecture, receptor expression, stromal density, and immune contexture precludes "one size fits all" nanomedicine efficacy. To address this challenge, the future of cancer nanomedicine -should shift from a one-size-fits-all model to personalized, precision therapies. Advances in multi-omics will enable detailed tumor profiling, guiding the design of nanocarriers with optimized properties for individual patients. AI and ML will analyze these profiles to predict nanoparticle behavior and identify biomarkers for patient stratification. Real-time monitoring via advanced imaging and biosensors will allow dynamic treatment adjustments, ensuring drug delivery is synchronized with the TME. Smart nanocarriers, capable of sensing and responding to biological cues, will enhance the precision of treatment. Biologically derived nanocarriers, such as exosomes, and microbiome-modulated nanomedicine will offer enhanced biocompatibility and targeting efficiency. In summary, the integration of precision medicine, AI, and real-time monitoring will lead to adaptive, patient-specific nanomedicine regimens that maximize efficacy and minimize toxicity, transforming cancer treatment [26,268].

### 7.2 Addressing multidrug resistance (MDR)

MDR remains a principal cause of treatment failure. Nanoscale co-delivery systems that simultaneously (i) silence efflux-pump genes (e.g., ABCB1, ABCG2) via RNAi, (ii) bypass P-gp-mediated extrusion through endocytic uptake, and (iii) release chemotherapeutics in a stimuli-gated manner, are poised to restore chemosensitivity [242,[269], [270], [271], [272]]. Integrating CRISPR-based gene editing within redox- or pH-responsive carriers further enables on-demand knock-down of resistance-conferring mutations, while preserving genomic integrity in healthy tissue [273].

### 7.3 AI-guided formulation and real-time theranostics

Next-generation cancer nanomedicine will be steered by AI frameworks that convert rich nanoparticle physicochemical descriptors into individualized PK–PD maps, generating digital twins capable of forecasting optimal dosing and flagging potential off-target accumulation in real time [22,[274], [275], [276]]. This vision is no longer purely speculative as the AI-assisted physiologically based pharmacokinetic (PBPK) model enhanced by ML already predicts tumor accumulation of specific nanomedicines in mice, providing an *in-silico* sandbox for formulation tuning before synthesis [274]. PRECISE CURATE.AI Pilot Trial (NCT04522284) uses the CURATE.AI platform to personalize chemotherapy (capecitabine-based) dosing via AI in advanced solid tumors [277]. Likewise, I-SPY (NCT01042379, NCT05868226) adaptively assigns therapies using multi-omic signatures, an algorithmic scaffold that can be transferred to nanocarrier selection based on tumor vascular permeability or immune landscape [278,279]. Furthermore, AI forecasts can be tethered to chip-to-cloud feedback, including ingestible capsules (IntelliCap) that stream luminal pH/temperature, implantable nano-biosensors that report intra-tumoral hypoxia or redox shifts, and microrobotic pill-injectors that dispense payloads on demand. These advancements have already improved the oral bioavailability of small-molecule drugs in early human studies [280,281]. The resulting closed-loop circuitry will shift clinical practice from static prescriptions to adaptive, self-optimizing regimens in which drug release is titrated to moment-by-moment physiological feedback. Cloud analytics will enable clinicians to refine dosing algorithms remotely, ensuring therapeutic exposure remains synchronized with evolving tumor biology while minimizing cumulative toxicities. Energy-efficient microchips and low-power wireless networks will sustain longitudinal tracking of both efficacy and patient adherence, enabling ultra-precise dose escalation or de-escalation [[282], [283], [284]]. While nanorobotics offers exciting prospects for precision drug delivery and *in situ* diagnostics, its clinical translation remains constrained by material, fabrication, and safety challenges. It requires rigorous elucidation of device biocompatibility, nanoparticle trafficking through dynamic biological barriers, and the mechanical biochemical interplay between micro-robotic systems and heterogeneous TME. Most prototypes rely on biocompatible metals (e.g., gold, iron oxide) or polymeric and DNA-based frameworks that are difficult to produce in mass with consistent functionality at the nanoscale. Fabrication methods such as self-assembly, lithography, and 3D nano-printing still face scalability and reproducibility limitations. Moreover, ensuring biodegradability, immune evasion, and safe clearance from the body are key safety bottlenecks yet to be fully addressed. Continued progress in bioinspired materials, soft robotics, and *in vivo* imaging is expected to gradually bridge these gaps toward clinically actionable, patient-calibrated cancer therapies [[285], [286], [287]].

### 7.4 Safe-by-design approaches

Despite tangible clinical benefits, nanomedicines can trigger unanticipated toxicities that elude conventional safety paradigms. Doxil®, the first FDA-approved nanotherapeutic, exemplifies this duality: while liposomal encapsulation reduced DOX-associated cardiotoxicity (0.78 % vs. 1.35 %) and myelosuppression (1.01 % vs. 1.33 %), it simultaneously introduced hypersensitivity reactions (1.78 %) and palmar–plantar erythrodysesthesia (4.37 %). These adverse events are mechanistically linked to prolonged circulation of PEGylated liposomes and the accumulation of negatively charged PEG-phosphatidylethanolamine conjugates in non-target tissues [[288], [289], [290]]. The field must transition from retrospective toxicology to prospective, safe-by-design frameworks to avert such liabilities. Nanomedicines reach patients only if their toxicological profile is predictable before first-in-human dosing. A tiered testing strategy *in-silico* modelling, *in-vitro* assays, *in-vivo* studies, and layered omics read-outs already supply mechanistic clues, quantitative structure activity/toxicity relationship (QSAR), and machine-learning models flag structure toxicity relationships [291], 2-D/3-D cell cultures quantify acute cytotoxicity and barrier integrity, rodent and non-rodent studies map biodistribution, metabolism, and long-term organ accumulation, transcriptomic, proteomic, and metabolomic signatures link these observations to pathway-level perturbations. Yet data dispersion, species discontinuities, and batch-to-batch variation still prevent the reliable ranking of the risk [292]. A curated, cross-species nanotoxicology database that harmonizes physicochemical descriptors with quantitative toxicokinetics and organ-specific long-term fate should be expanded. Coupling these resources with high-throughput organ-on-chip arrays, single-cell multi-omics, and AI-driven predictive models will enable *in-silico* risk stratification before *in-vivo* evaluation. At the same time, quality-by-design (QbD) principles and real-time release analytics ensure batch-to-batch consistency. Significantly, safe-by-design extends beyond the carrier itself; co-formulation with immunotherapeutics, gene editors, or neoantigen vaccines mandates orthogonal safety assays that interrogate immune priming, off-target editing, and sustained immune activation. The synergistic integration of these datasets will yield nanomedicines that deliver drugs with precision and carry an intrinsically lower risk profile [291,293].

### 7.5 Optimization and precision of tumor targeting

Ligand-mediated nanomedicine promises molecular-level selectivity, yet multifactorial obstacles constrain its clinical realization [18]. Optimal targeting demands ligands that exhibit high affinity for tumor-restricted receptors while evading immune surveillance; achieving this balance is complicated by inter-patient heterogeneity in antigen expression and glycosylation patterns. Once bound, cellular internalization efficiency is dictated by a complex interplay among ligand density, nanoparticle geometry (aspect ratio and curvature), surface charge, and local shear forces within the TME. An additional confounder is the rapid formation of a biomolecular corona of proteins, lipids, and nucleic acids that adsorb onto the nanoparticle surface, potentially shielding targeting moieties and redirecting biodistribution. To overcome these hurdles, next-generation strategies should deploy dynamic surface chemistries: zwitterionic or glycan shields that suppress non-specific protein adsorption, pH-cleavable PEG layers that expose ligands only within the acidic TME, and ML-guided libraries that optimize ligand valency and orientation for maximal endocytic uptake [[294], [295], [296], [297], [298]]. CRISPR-engineered cell lines and precise patient-derived organoids will serve as high-fidelity testbeds for iterative ligand refinement, while AI-driven design loops will predict corona-resistant architectures. Ultimately, the convergence of genetic barcoding, single-cell intravital imaging, and adaptive surface engineering will yield nanomedicines that achieve molecular-scale precision with macro-scale therapeutic impact [27,276,[299], [300], [301], [302]].

## 8 Conclusion

Cancer nanomedicine stands at a pivotal juncture, poised to transition from a promising platform to the cornerstone of precision oncology. The evolution from passive carriers to smart, multi-stimuli-responsive systems has been profound, yet the true paradigm shift lies in personalization. By harnessing AI to decode patient-specific multi-omics profiles into actionable nanocarrier designs, we can finally convert tumor heterogeneity from a barrier into a therapeutic blueprint. Overcoming the translational challenges demands a convergent, proactive strategy: embracing Safe-by-Design principles, establishing scalable Quality-by-Design manufacturing, and implementing harmonized regulations. The integration of these elements with real-time biosensing and closed-loop feedback will ultimately transform nanomedicine into an adaptive, patient-calibrated therapeutic engine, delivering on the long-held promise of precision cancer therapy.

## CRediT authorship contribution statement

**Ayesha Younas:** Writing – review & editing, Writing – original draft, Visualization, Software, Methodology, Funding acquisition, Conceptualization, Formal analysis. **Shuanghu Wang:** Resources, Funding acquisition, Data curation. **Muhammad Asad:** Validation, Investigation, Data curation. **Abdullah Al Mamun:** Formal analysis. **Saadat Majeed:** Investigation, Formal analysis. **Ali Sharif:** Investigation, Formal analysis. **Quan Zhou:** Formal analysis. **Yunxiao Liu:** Formal analysis. **Peiwu Geng:** Formal analysis. **Chuxiao Shao:** Supervision, Resources, Funding acquisition. **Jian Xiao:** Validation, Supervision, Project administration, Investigation, Conceptualization, Resources.

## Declaration of competing interest

The authors declare that they have no known competing financial interests or personal relationships that could have appeared to influence the work reported in this paper.

## Data Availability

No data was used for the research described in the article.
